# Image-guided drug delivery of nanotheranostics for targeted lung cancer therapy

**DOI:** 10.7150/thno.72803

**Published:** 2022-05-13

**Authors:** Xiaoran Yin, Yanan Cui, Richard S. Kim, Wesley R. Stiles, Seung Hun Park, Haoran Wang, Li Ma, Lin Chen, Yoonji Baek, Satoshi Kashiwagi, Kai Bao, Amy Ulumben, Takeshi Fukuda, Homan Kang, Hak Soo Choi

**Affiliations:** 1 Department of Oncology, The Second Affiliate Hospital of Xi'an Jiaotong University; Xi'an, Shaanxi, 710004, China; 2 Gordon Center for Medical Imaging, Department of Radiology, Massachusetts General Hospital and Harvard Medical School; Boston, MA 02114, USA; 3 School of Pharmacy, Jining Medical College; Rizhao, Shandong, 276826, China; 4 Department of Pathology, The Second Affiliate Hospital of Xi'an Jiaotong University; Xi'an, Shaanxi, 710004, China; 5 Department of Pathology, Shaanxi Province People's Hospital, Xi'an; Shaanxi, 710068, China

**Keywords:** Theranostics, Cancer therapy, Image-guided surgery, Combination therapy, Renal clearance

## Abstract

Enormous efforts have been made to integrate various therapeutic interventions into multifunctional nanoplatforms, resulting in the advance of nanomedicine. Image-guided drug delivery plays a pivotal role in this field by providing specific targeting as well as image navigation for disease prognosis.

**Methods:** We demonstrate image-guided surgery and drug delivery for the treatment of lung cancer using nanotheranostic H-dots loaded with gefitinib and genistein.

**Results:** The surgical margin for lung tumors is determined by image guidance for precise tumor resection, while targeted anti-cancer drugs function simultaneously for synergistic combination therapy. Compared to conventional chemotherapies, H-dot complexes could improve the therapeutic efficacy of drugs while reducing the risk of adverse effects and drug resistance owing to their ideal biodistribution profiles, high targetability, low nonspecific tissue uptake, and fast renal excretion.

**Conclusions:** These H-dot complexes have unlocked a unique framework for integrating multiple therapeutic and diagnostic modalities into one nanoplatform.

## Introduction

Lung cancer remains the leading cause of cancer-related deaths worldwide, and the 5-year survival rate for patients with metastatic non-small cell lung cancer (NSCLC) has been very low (< 5%) until the last decade 1, 2. Unfortunately, almost 74% of lung cancer cases are diagnosed at an advanced stage (locally advanced stage or metastatic stage). Cases at previous stages with locally advanced tumors (earlier than stage ⅢA) can potentially be cured by surgery 3. However, some NSCLC patients at stage IB with risk factors, stage Ⅱ, or stage ⅢA are highly prone to recurrence and poor prognoses even after complete curative surgical resection due to micrometastasis 4. To improve the survival rates and outcomes of these patients, a new perioperative therapeutic strategy that employs treatments with multifunctional nanoparticles before and after surgical resection (i.e., neoadjuvant/adjuvant chemotherapy) has been developed, which is helpful for controlling micrometastasis and reducing the risk and difficulty of surgical resection. In this context, theranostic nanoplatforms that provide both therapeutic and surgical interventions have recently received tremendous attention as the next generation of targeted therapies 5-7.

Advances in targeted therapy of malignant lung tumors have been led by the development of epidermal growth factor receptor tyrosine kinase inhibitors (EGFR-TKIs) which are widely applied in the clinic as the standard first-line option for treating EGFR-mutant NSCLC with category 1 recommendations 8. Gefitinib (Gef) induces apoptosis by targeting the ATP cleft to prevent EGFR autophosphorylation 9 and has a high clinical benefit 10. Compared to those treated with traditional chemotherapy, Gef treatment in EGFR-mutated NSCLC patients during the perioperative period has improved survival rates 10-12. However, most patients inevitably develop acquired resistance to Gef within 8 to 12 months 13, and Gef can lead to interstitial lung disease (ILD) and acute/chronic liver injury 14. In order to improve the outcomes of lung cancer patients, combinations of EGFR-TKI with different drugs (including other TKIs, monoclonal antibodies, and chemotherapy agents) have been intensively investigated 15. Genistein (Gen) is a soy-derived isoflavone and phytoestrogen which exhibits potent antiangiogenic properties via downregulation of the transcription of pro-angiogenic factors such as vascular endothelial growth factor (VEGF) 16. Angiogenesis inhibitors (AIs) normalize tumor vasculature which will improve tumor perfusion, uptake of anti-tumor drugs, and efficacy of tumor therapies 17, 18. In addition, AIs have been added to EGFR-TKI treatments to dual-blockade the VEGF/EGFR pathways in EGFR-mutant NSCLC preclinical models resulting in the reversal of primary or acquired resistance to EGFR-TKIs 17, 19-24.

Incomplete dissection or positive surgical margins results in recurrence and metastases in patients 25, 26. Although endobronchial ultrasound-guided transbronchial needle aspiration (EBUS-TBNA) and positron emission tomography (PET)/computed tomography (CT) have been used for the preoperative evaluation of tumors 27, it is difficult to localize and determine a safe margin for tumors and/or lymph node metastasis (LNM) that may not be visible to the naked eye during surgery. Therefore, intraoperative guidance of the tumor and LNM is essential for complete and safe excision 28. Consequently, it is in high demand to develop novel and reliable strategies which enable precise identification of tumors during complex surgical interventions, a task for which real-time near-infrared (NIR) fluorescence imaging is well-equipped.

**Scheme 1:**
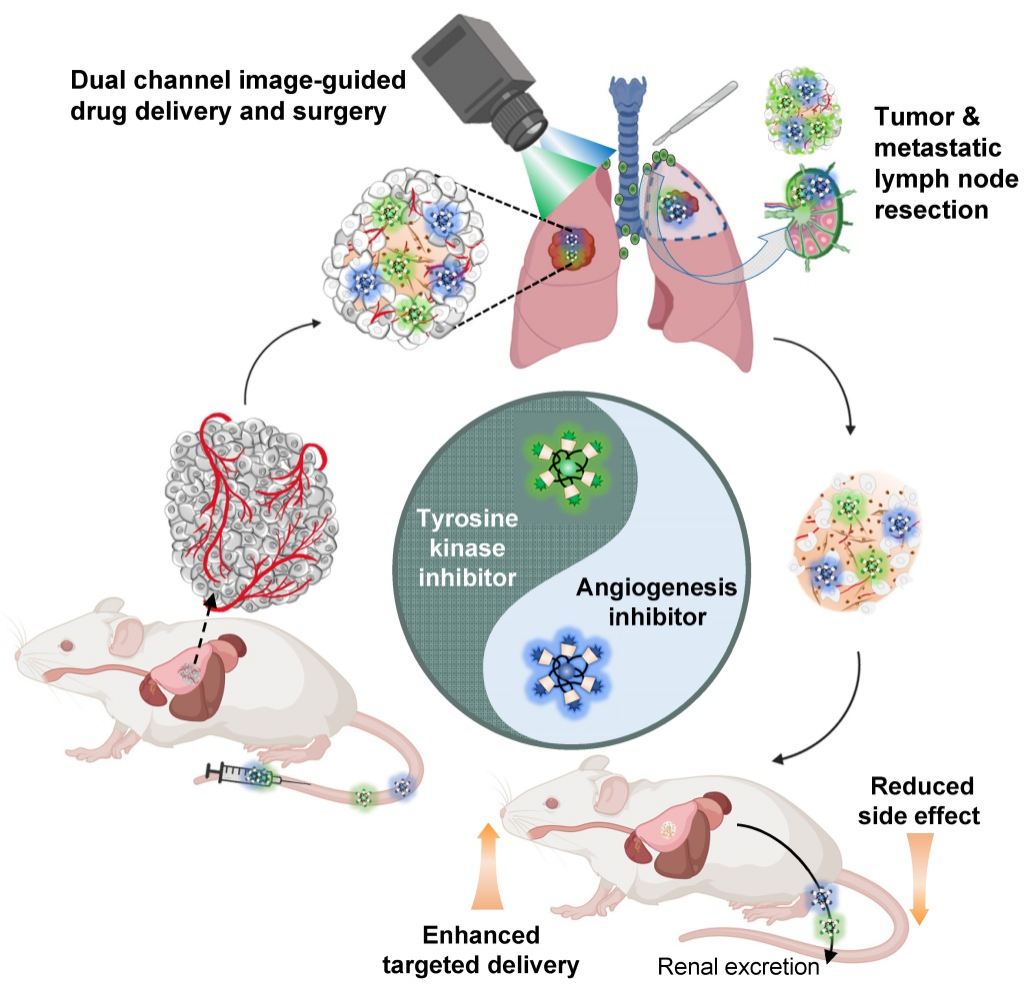
** Combination therapy of EGFR-tyrosine kinase inhibitor (EGFR-TKI) and angiogenesis inhibitor (AI) incorporated in nanotherapeutic H-dot.** The multifunctional H-dot complexes with TKI and AI enable dual-channel NIR fluorescence image-guided surgical intervention in real-time as well as transportation of targeted drugs (i.e., TKI and AI) for synergistic combination therapy. The H-dot-based targeted drug delivery system is renal clearable and nonsticky, which reduces the side effects of anti-cancer target drugs while improving therapeutic efficacy. This is a new strategy for synthesizing multifunctional theranostic agents as one nanoplatform for precise diagnosis and targeted therapy of lung cancer.

In this study, we demonstrated the combination of image-guided lung cancer/LNM surgery and targeted combination therapy by using the multifunctional theranostic nanoplatform, H-dots (Scheme 1). The Gef/H-dot and Gen/H-dot complexes not only provided real-time dual-channel intraoperative NIR fluorescence image guidance and pathological assistance but also delivered both EGFR-TKI (Gef)/AI (Gen) simultaneously to the tumor sites for synergistic treatment with low nonspecific uptake by normal tissues to reduce adverse effects in orthotopic lung tumor and subcutaneous tumor mice models. In addition, the drug release (Gef and Gen) in the acidic tumor microenvironment and the synergistic effects of Gef/H-dot and Gen/H-dot complexes were confirmed. This innovative, noninvasive approach to drug delivery is a promising targeted theranostic tool with specific biodistribution, better water solubility and bioavailability, as well as rapid excretion from the body.

## Results

### Design, synthesis, and characterization of H-dot

**Figure 1:**
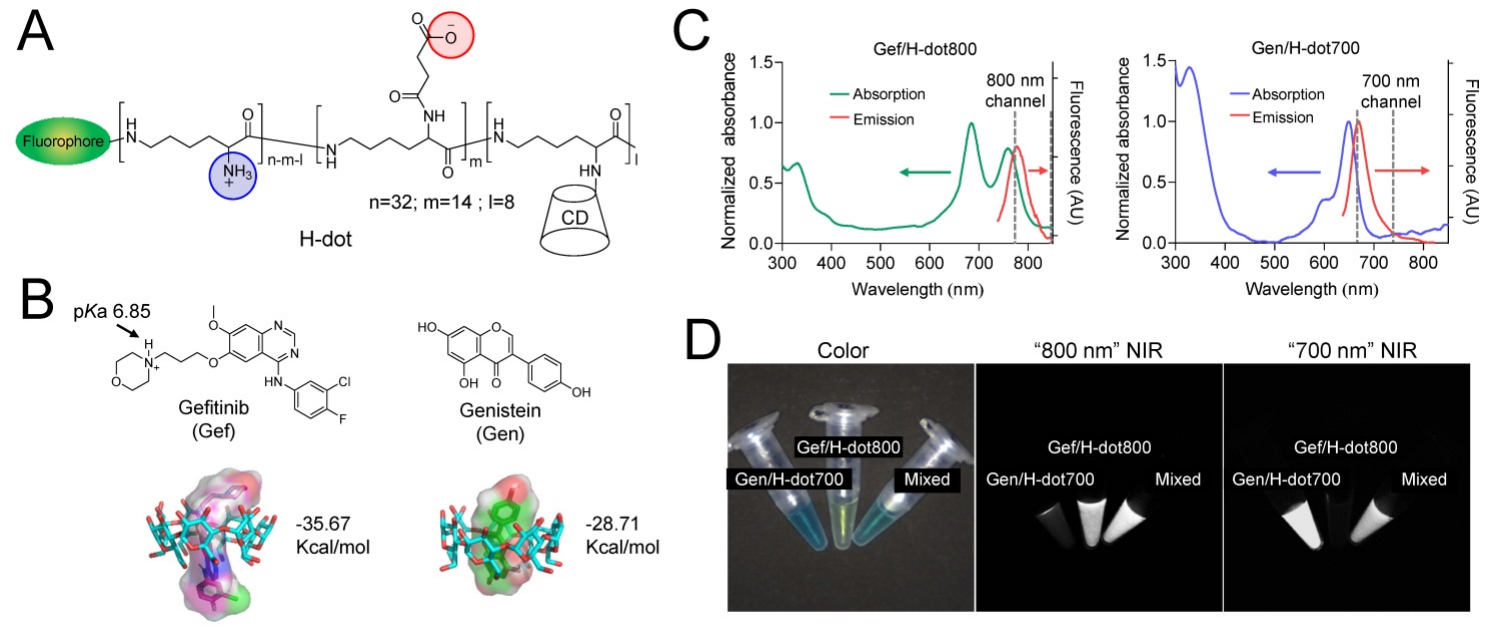
**Physicochemical properties of theranostic H-dot complexes. (A)** Chemical structure of H-dot. **(B)** Chemical structures of Gen and Gef and their inclusion complexes with beta-cyclodextrin (CD). The numbers indicate binding energies using the CDOCKER protocol in Discovery Studio 3.0 software. **(C)** Absorption and fluorescence emission spectra of Gen/H-dot700 and Gef/H-dot800 complexes. **(D)** Dual-channel NIR fluorescence images (700 nm and 800 nm channels) of the Gen/H-dot700, Gef/H-dot800, and their mixture.

The nanotheranostic H-dot is composed of biocompatible ε-poly-L-lysine (EPL) and β-cyclodextrin (CD) and is designed for delivering hydrophobic drugs. H-dot has several key features for the targeting and therapy of lung cancer: 1) The zwitterionic surface of the H-dot imparts the ability to surpass biological barriers such as the mononuclear phagocyte system (MPS). 2) The size of the H-dot is smaller than the kidney filtration threshold which allows it to be excreted through the kidneys to the urinary bladder, 3) but is large enough to maintain tumor targetability via the small size enhanced permeability and retention (EPR) effect 29. 4) H-dots include a NIR fluorescent moiety which enables real-time intraoperative imaging, monitoring of tumor targeting, pharmacokinetics, and drug delivery following precise surgical resection 25, 30. H-dots were synthesized following a previously reported synthetic route 25, 31 which is detailed in Supporting Information. To confirm the structure, the H-dots were characterized thoroughly using several techniques. The hydrodynamic diameter (HD) was determined by performing size-exclusion chromatography and comparing the retention volume of H-dots and CD conjugated EPL (CDPL^+^), a positively charged H-dot precursor, to a protein standard curve (Figure S1 and S2, Supporting Information). CDPL^+^ was shown to have an HD of 4.93 nm, whereas the final H-dot was measured to be 5.75 nm in diameter. Interestingly, the hydrodynamic diameter (HD) of Gef/H-dot and Gen/H-dot was found to be 5.84 nm and 5.85 nm, respectively, similar to that of H-dot alone (5.75 nm), resulting from the low loading capacity (mass of encapsulated drug/total mass of nanoparticles ×100) of Gef (4.9%) and Gen (3.4%), respectively (Figure S1 and S6, Supporting Information). To evaluate the surface charge of H-dots, we employed the ninhydrin test and UV-Vis spectroscopy to determine that 59% of the primary amines (+ charge) had been converted to carboxylates (- charge), imparting a near zwitterionic surface onto the final H-dot (Figure S3, Supporting Information). Through ^1^H-NMR spectroscopy, it was shown that 8.1 CD moieties on average were grafted onto the EPL backbone (Figure S4, Supporting Information). Using these characterizations, an accurate structure of H-dot was elucidated (Figure 1A and Figure S5, Supporting Information). The size and surface characteristics are the key components for the design of theranostic nanoplatforms. These factors influence serum binding, biodistribution, pharmacokinetics/pharmacodynamics (PK/PD), targetability, and therapeutic efficacy and toxicity.

### Preparation of drug/H-dot complexes and drug release test

**Figure 2:**
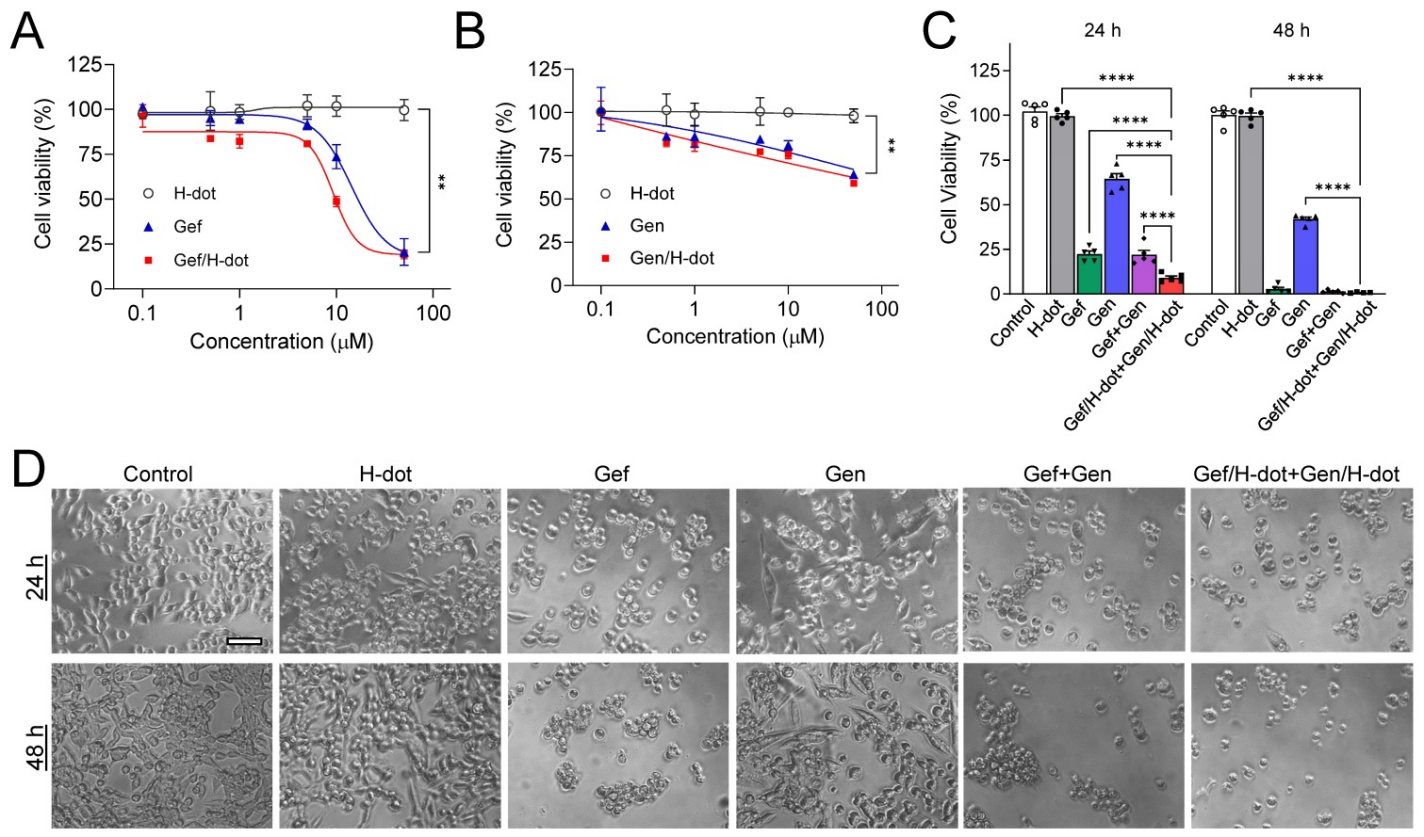
***In vitro* therapeutic efficacy test of Gef/H-dot and Gen/H-dot complexes. (A)** LLC cell growth inhibitory effects at 24 h with different concentrations of H-dot, Gef, and Gef/H-dot complex, and **(B)** H-dot, Gen, and Gen/H-dot complex treatment group at 24 h. (n = 5, mean ± s.e.m.). **(C)** LLC cell growth inhibitory effect with H-dot800 (40 µM) +H-dot700 (30 µM), Gef (40 µM), Gen (30 µM), Gef+Gen (40 µM + 30 µM), and Gef/H-dot (40 µM) with Gen/H-dot (30 µM) at 24 h and 48 h (n = 5, mean ± s.e.m.). *p* values < 0.05 were considered significant: **p < 0.01 and ****p < 0.0001. **(D)** phase-contrast microscope images (×20) to compare the morphology and confluency of LLC cells after treatment with H-dot, Gef, Gen, Gef+Gen, and Gef/H-dot+Gen/H-dot for 24 h and 48 h. Scale bar: 200 µm.

To deliver EGFR-TKIs and AIs and monitor the delivery of each drug simultaneously in the body, Gef and Gen were loaded into 800 or 700 nm fluorescence emitting dye-conjugated H-dots (denoted as H-dot800 and H-dot700), respectively. The loaded amount of Gef and Gen in H-dots was determined by the UV absorbance increase at 332 nm and 258 nm, respectively, and the molar ratios of Gef and Gen to H-dot were calculated as 1.8 and 2.0, which correspond to encapsulation efficiencies of 90% and 36% for Gef and Gen, respectively (Figure S6, Supporting Information). This result indicates that Gef forms an inclusion complex more efficiently with H-dots compared to Gen. There were no solubility issues of drug/H-dot complexes, even at very high concentrations (~0.5 M), indicating that H-dots can increase the water solubility of hydrophobic drugs greatly through the formation of a stable inclusion complex. In addition, a docking simulation was conducted using the CDOCKER protocol in Discovery Studio 3.0 which showed low binding energies of Gef and Gen complexed with CD, which were -35.67 and -28.71 kcal mol^-1^, respectively, suggesting that inclusion complexes are quite stable in physiological conditions (Figure 1B). After making inclusion complexes, the optical properties of Gef/H-dot800 and Gen/H-dot700 were obtained using UV-Vis spectroscopy and our dual-channel fluorescence imaging system (K-FLARE) (Figure 2C-D). The maximum wavelengths of the fluorescence emission peaks for Gef/H-dot800 and Gen/H-dot700 were 775 and 670 nm, respectively. Therefore, Gef/H-dot800 was visualized in the 800 nm channel, and Gen/H-dot700 was visualized in the 700 nm channel. This dual-channel imaging allows us to track the TKI(Gef)/H-dot complex in the 800 nm NIR channel and the AI(Gen)/ H-dot complex in the 700 nm NIR channel.

Before assessing* in vitro* anti-tumor efficacy, the pH-responsive drug release of Gef/H-dot and Gen/H-dot was tested in different pHs (6.0 and 7.4) at 37 ^º^C for 8 h (Figure S7, Supporting Information). Released Gef or Gen from the inclusion complexes was determined by measuring UV absorption. In the Gen/H-dot release test, about 40% of Gen was rapidly released within 8 h post-incubation at pH 6.0. In contrast, around 70% of Gef was released from the Gef/H-dot complex at pH 6.0 compared to 49% at pH 7.4. This is due to the monoprotonation of the cyclic tertiary amine (p*K*a 6.85) of Gef at pH 6.0 (comparable to the pH of the tumor microenvironment) resulting in the disruption of the hydrophobic interaction between Gef and the nonpolar cavity of β-CD, causing release (Figure 1B). These results suggest that the release of Gen from the Gen/H-dot complex is dependent only on time, while the release of Gef from the Gef/H-dot complex is dependent on the pH of the surrounding environment, with greater release occurring in acidic tumor microenvironments.

### In vitro evaluation of synergistic anti-tumor therapeutic efficacy

*In vitro* therapeutic efficacy and kinetics of Gef/H-dot and Gen/H-dot complexes were evaluated in LLC cells with various concentrations (0.1, 0.5, 1, 5, 10, and 50 µM, all concentrations of drug/H-dot are with respect to the H-dot, see Table S1, Supporting Information, for the detailed dose information) by CCK-8 (cell counting kit-8) assays and microscopy, and the results were compared to those of H-dot, Gen, and Gef alone (Figure 2A-B and Figure S8A, Supporting Information). In the H-dot alone group, there was no significant evidence of cytotoxicity or cell morphological changes as compared to the negative control group, even at the highest dose (50 μM), indicating that H-dot is safe for further *in vivo* applications. In contrast, the Gef/H-dot or Gen/H-dot treated group clearly showed dose-dependent cell deaths at 24 h post-treatment (**p <0.01; Figure 2A-B), following a similar pattern to the free Gef or Gen treatment, respectively. By using CompuSyn software, the combination index (CI) of Gef+Gen was calculated to be 0.34232, a value which indicates “Synergism” (Figure S8B). It is important to note that Gef and Gef/H-dot treatment exhibited strong cytotoxicity higher than 10 µM, and the half-maximal effective concentration (EC50) for Gef/H-dot was calculated to be 9.28 µM which is better than Gef alone (14.40 µM) (Figure 2C).

Next, the synergistic therapeutic efficacy was evaluated by treatment with Gef/H-dot+Gen/H-dot complexes. Due to the poor solubility of native Gef and Gen in aqueous media, the treated dose of Gef/H-dot+Gen/H-dot was set as 40 µM Gef/H-dot, and 30 µM Gen/H-dot, based on the maximum solubility of the drugs themselves (Table S1, Supporting Information). The Gef/H-dot+Gen/H-dot combination showed much higher therapeutic efficacy than other treatment groups even compared with Gef alone or Gef+Gen groups at 24 h (***p < 0.0001, Figure 2C). The decreased cell density, changes in cell morphology, and loss of cell adhesion and increased floating were observed in the Gef/H-dot+Gen/H-dot group (Figure 2D). The combination of Gef/H-dot+Gen/H-dot complexes showed a synergistic anti-tumor therapeutic efficacy, greater than that of either Gef or Gen alone at 24 h due to the ability of H-dots to efficiently deliver target drugs to tumor cells with better performance characteristics than free drugs. These results confirmed the promising application of the drug/H-dot complexes to the synergistic treatment against lung tumor cells.

### In vivo biodistribution and pharmacokinetics of drug/H-dots

**Figure 3:**
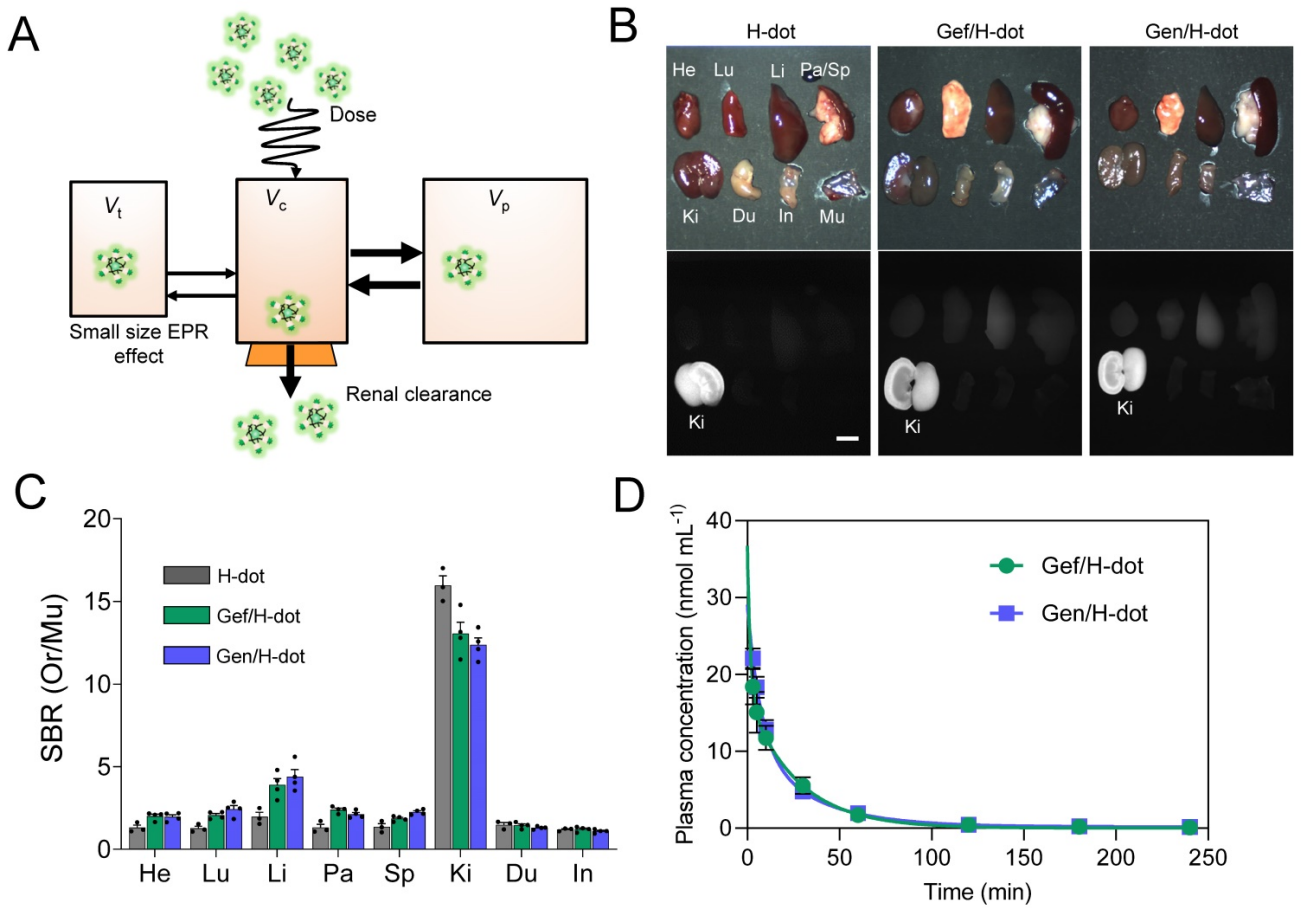
** Biodistribution and pharmacokinetics for Gef/H-dot and Gen/H-dot.** Drug/H-dot complexes were injected into CD-1 mice, and NIR imaging was carried out at 4 h post-injection. **(A)** Schematic diagram for pharmacokinetics/dynamics, distribution, and clearance of renal clearable H-dot. *V*_t_, *V*_c_, and *V*_p_ stand for volume of the tumor, volume of the central compartment, and volume of the peripheral compartment, respectively. **(B)** Color and NIR fluorescence images of resected organs. Abbreviations used are: Du, duodenum; He, heart; In, intestine; Ki, kidneys; Li, liver; Lu, lungs; Mu, muscle; Pa, pancreas; Sp, spleen. Scale bar: 5 mm. **(C)** Signal to background ratio (SBR) of each resected organ from mice injected with H-dot, Gef/H-dot, and Gen/H-dot. (n = 3-4 per group, mean ± s.e.m.) **(D)** Plasma concentration decay curve of Gef/H-dot and Gen/H-dot. Blood samples from Gef/H-dot and Gen/H-dot injected mice were collected at time points: 1, 3, 5, 10, 30, 60, 120, 180, and 240 min. (n = 4 per group, mean ± s.e.m.)

Known for its superior renal clearable ability, H-dot has proved to be the ideal drug nanocarrier for tumor targeting and drug delivery for gastrointestinal stromal tumors (GIST) 25, 31. In this section, we evaluated the possibility of using H-dot as a carrier for Gef and Gen co-deliveries aiming to increase tumor accumulation/therapeutic efficacy and reduce the adverse effects of the drugs (Figure 3A). Firstly, the biodistribution of the Gef/H-dot and Gen/H-dot were investigated. The two complexes showed almost the same accumulation in all the organs and could be cleared through the kidney (Figure 3B and C and Figure S9, Supporting Information). For the two complexes, the signal-to-background ratio (SBR) in the heart, lung, liver, pancreas, and spleen was slightly higher than that of H-dot only. This can be attributed to the increased hydrophobicity of drug/H-dots due to the inclusion of Gef and Gen, which lead to a small increase in nonspecific distribution.

To further investigate the in vivo characteristics of the drug/H-dot complexes, the pharmacokinetic properties of Gef/H-dot and Gen/H-dot were examined after a single intravenous injection and compared with H-dot alone (Figure 3D, Table S2, and Figure S10, Supporting Information). The NIR fluorescent signal intensity from mouse serum collected at predetermined time points was measured to obtain the blood concentration decay curves of the drug/H-dot complexes. The results demonstrated that both Gef/H-dot and Gen/H-dot complexes exhibited pharmacokinetic behavior consistent with the two-compartment model. The drug/H-dot complexes distributed rapidly into major organs (*t*_½α_ = 9.08 ± 10.37 and 5.85 ± 2.01 min for Gef/H-dot and Gen/H-dot, respectively) and then eliminated quickly from the body (*t*_½β_ = 23.87 ± 4.09 and 28.72 ± 5.74 min for Gef/H-dot and Gen/H-dot, respectively) with a fast plasma clearance rate (0.124 mL·min^-1^). The values for the volume of distribution (186 mL·kg^-1^ for Gef/H-dot and 207 mL·kg^-1^ for Gen/H-dot) are similar to the volume of the extracellular fluids (~200 mL·kg^-1^), indicating that the two complexes distributed into the whole body without specifically sticking to the peripheral compartment. These results suggest that drug/H-dots experience nontoxic events in the body and are conducive to effective renal clearance.

Next, we conducted a quantitative analysis of tumor targeting and drug delivery efficiency for Gef/H-dot and Gen/H-dot by using NIR imaging and HPLC of resected tumor tissue at 24 h post-injection of Gef/H-dot+Gen/H-dot (Figure S11, Supporting Information). Tumor targetability (%ID g^-1^) of the drug/H-dot was calculated to be 1.82 ± 0.01 %ID g^-1^ (15 µmol kg^-1^, IV injection) for drug/H-dot in the tumor at 24 h post-injection (data was not shown). In addition, HPLC showed that 6.2 and 0.7 %ID/g were delivered for Gef and Gen, respectively. The %ID/g of Gef is higher than that of H-dot itself (1.82 %ID/g), which indicates H-dot was able to efficiently deliver and release the hydrophobic drug at low pHs, such as in the tumor environment. Then, H-dot likely cleared out from the tumor tissue slowly. However, the %ID/g of Gen is relatively low which could be attributed to its low pH response and stability.

### In vivo real-time image guidance for lung and metastatic lymph node resections

**Figure 4:**
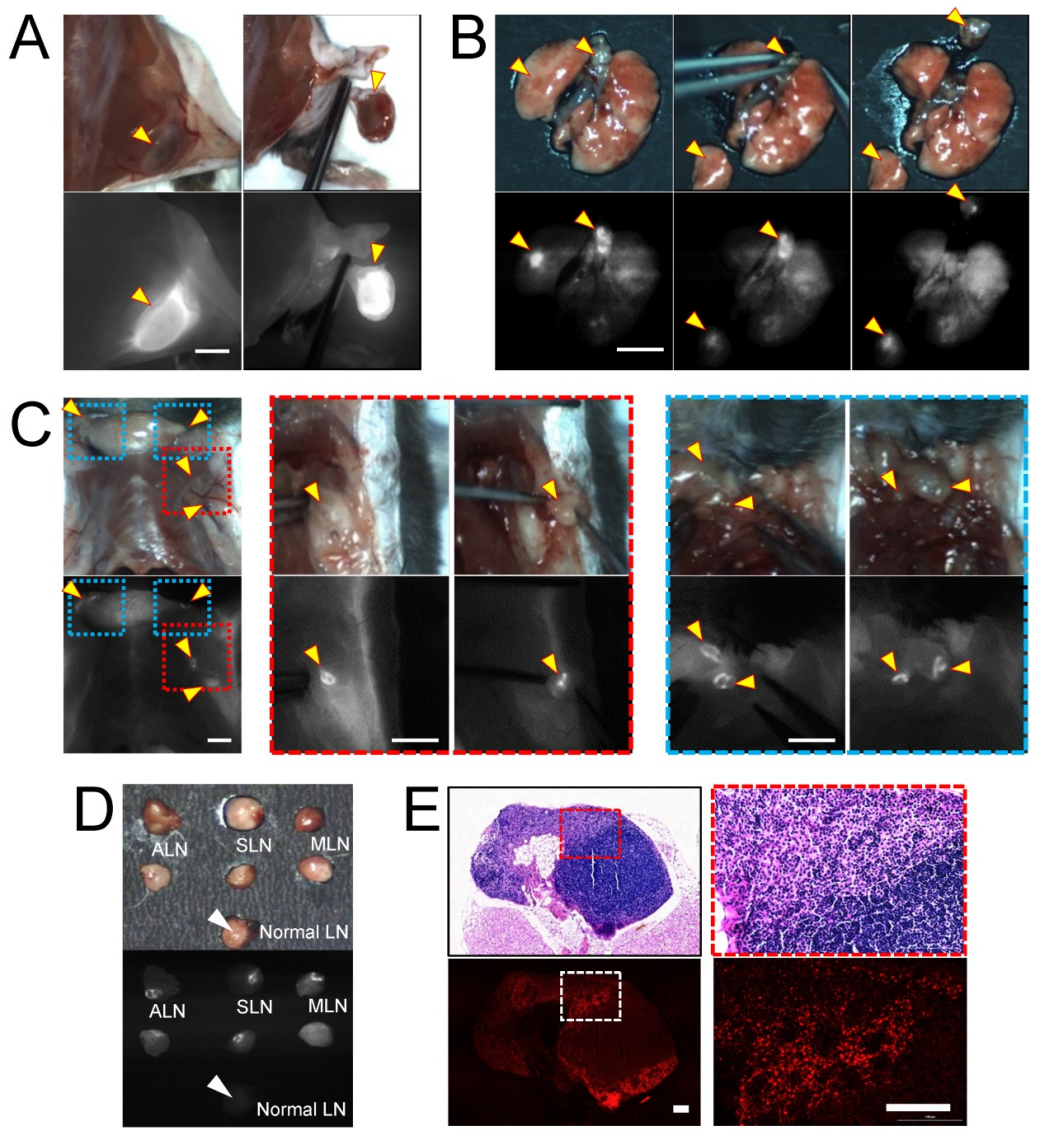
***In vivo* real-time image-guided surgical intervention and NIR fluorescence-guided histopathology test. NIR fluorescence imaging of LLC lung tumor-bearing mice at 24 h post-injection of Gef/H-dot. (A)** NIR fluorescence image-guided surgery of subcutaneous tumor, **(B)** orthotopic lung tumor and mediastinal metastatic lymph nodes, and **(C)** regional/distant metastatic lymph nodes including axillary (red dotted square outline) and supraclavicular (blue dotted square outline) lymph nodes metastases. Scale bar: 5 mm. Yellow arrowheads indicate tumors and metastatic lymph nodes which were removed under real-time image guidance. **(D)** Resected metastatic lymph nodes compared to normal lymph nodes under the guidance of intraoperative fluorescence imaging, **(E)** postoperative histopathological examination; H&E staining images (upper panel) and NIR fluorescence microscopic images (lower panel). LLC tumor cells were able to be distinguished from lymphocytes. Scale bar: 100 µm.

To evaluate the imaging capacity of H-dots for intraoperative guidance, we injected Gef/H-dot into either subcutaneous or orthotopic LLC lung cancer mouse models and observed the fluorescence signal of Gef/H-dot in the mice using K-FLARE at 24 h post-injection (Figure 4). In the subcutaneous lung cancer model, the cancerous region showed a high NIR fluorescent SBR and could be easily dissected based on the fluorescence image guidance (Figure 4A). Next, a lung segmentectomy and local metastatic lymph node resections in the orthotopic lung tumor mouse model were performed to demonstrate the resections of small-sized tumors (less than 5 mm, yellow arrowheads in Figure 4B), which reflects the natural environment of primary lung tumors and pulmonary metastatic tumors. It is worth noting that strong fluorescence was found in regional mediastinal lymph nodes and regional/distant lymph nodes including the axillary (red dotted line squares in Figure 4C) and supraclavicular (blue dotted line square in Figure 4C) lymph nodes in the orthotopic lung tumor model mice. Aided by fluorescence image guidance, lymph node resections and postoperative pathological analysis were performed (Figure 4C-E and Movie S1, Supporting Information) to prove the lymph nodes were metastatic rather than a false-positive. Metastatic lymph nodes have significantly higher NIR fluorescent signals compared with normal lymph nodes (Figure 4D). Metastatic tumor cells were clearly identified from normal lymphocytes in H&E staining images (red dotted line square) and NIR fluorescence microscopic images (white dotted line square). Also, the boundaries between metastatic tumor cells and normal lymphocytes were consistent among the H&E staining image and the NIR fluorescence microscopic image (Figure 4E and Figure S12, Supporting Information). These results suggest that the Gef/H-dot complex targets only tumor cells, not normal cells. Moreover, it will assist pathologists in distinguishing tumor areas and metastatic lymph nodes quickly and accurately without histological staining. Due to the excellent targetability of the H-dot complex, subcutaneous lung tumors, orthotopic lung tumors, and even metastatic tumors of the lymph nodes were resected with narrow margins through image-guided surgery. These results indicate that the H-dot complex has a suitable targetability for small-sized tumors such as metastatic tumors as well as massive tumors. Importantly, there exists a great possibility to apply H-dots to the identification of metastatic tumors that lead to around 90% of cancer-related mortalities in the clinic.

### In vivo synergistic combination therapeutic efficacy of the Gef/H-dot800 and Gen/H-dot700 complexes

**Figure 5:**
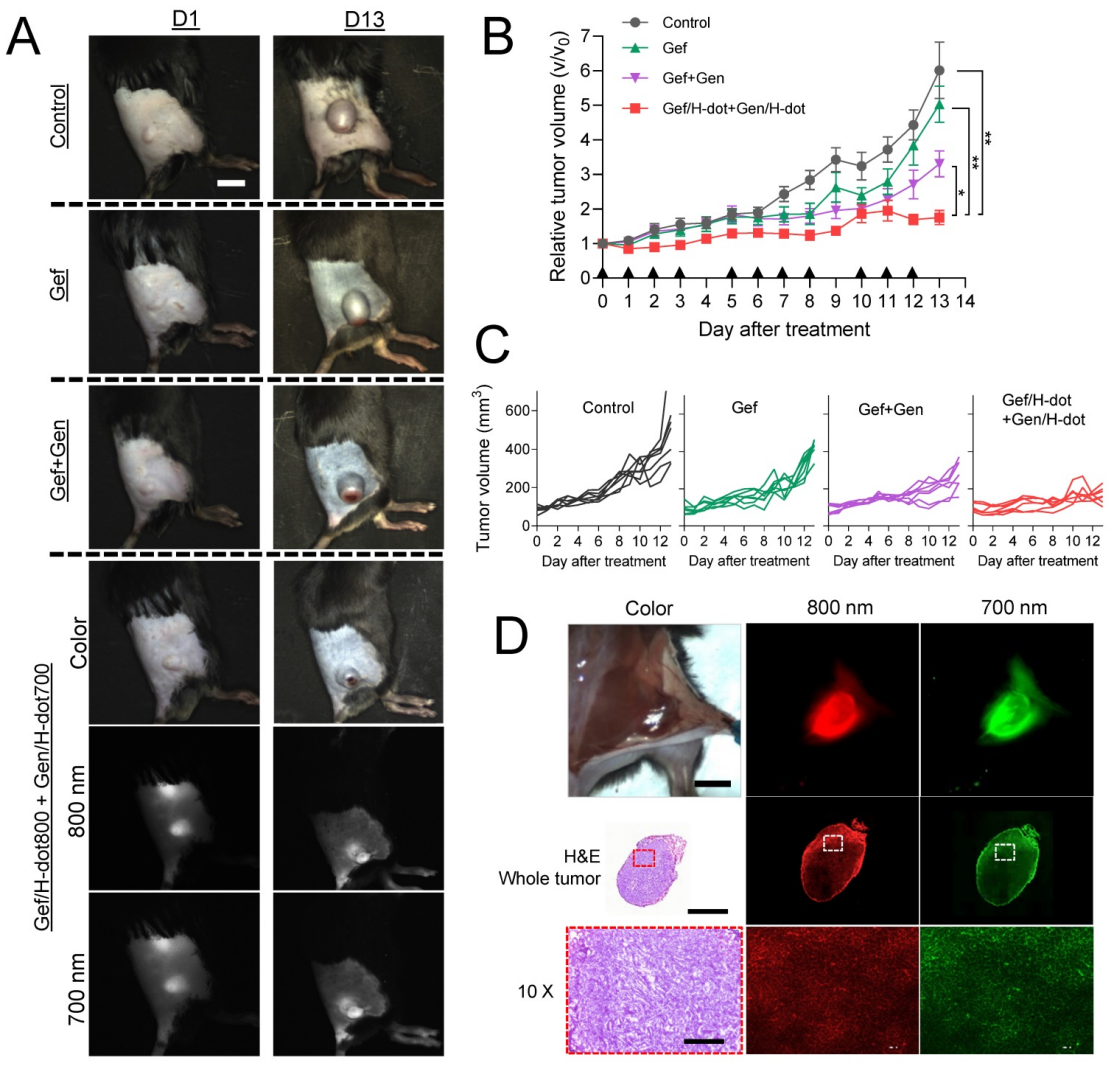
***In vivo* therapeutic efficacy of combination treatment with theranostic Gef/H-dot and Gen/H-dot compared to saline, Gef, and Gef+Gen groups for D13 treatment (*n* = 7 per each group). Black arrowheads indicate the day of injections. (A)** Representative color and NIR images of each treatment group on D1 and D13. Scale bar: 5 mm. **(B)** Tumor growth curves for the mice of the four different treatment groups. *p* values < 0.05 were considered significant: *p < 0.05 and **p < 0.01. **(C)** Individual tumor volume for each treatment group. **(D)** Intraoperative NIR fluorescence and microscope images of a resected tumor after treatment with the combination of Gef/H-dot800 and Gen/H-dot700. Scale bar: 5 mm, 2 µm, and 200 µm for intraoperative imaging, whole tumor, and 10× microscope images, respectively.

Encouraged by the superb *in vitro* synergistic efficacy, biodistribution, and pharmacokinetic results of the drug/H-dot complexes, the *in vivo* anti-tumor efficacy of those complexes was evaluated. LLC lung cancer-bearing mice were treated with four different formulations: saline, Gef, Gef+Gen, and Gef/H-dot800+Gen/H-dot700 (n = 7 per each group; see Table S3, Supporting Information, for the detailed doses). The treatment lasted 13 d and was given 11 times as scheduled, and the mice were monitored every day using our dual-channel NIR imaging system to assess the therapeutic effects of each treatment. Figure 5A shows the tumor sizes and dual NIR signals (800 nm and 700 nm channels) of accumulated Gef/H-dot+Gen/H-dot in cancerous regions on D1 and D13. It was confirmed that Gef/H-dot+Gen/H-dot treatments significantly suppressed tumor sizes overtime (***p < 0.0001 compared to saline and Gef groups, *p < 0.05 compared to Gef+Gen group) and showed excellent tumor-to-background ratios without nonspecific adsorptions (except kidneys) even after repeated injections (Figure 5A, B, and D). Interestingly, the Gef, Gef+Gen, and Gef/H-dot+Gen/H-dot groups showed similar tumor suppressions compared to the saline control group until D10, after which the tumor growth rate accelerated in the Gef and Gef+Gen treatment groups as well as the control group, while tumor growth was suppressed in the Gef/H-dot+Gen/H-dot group (Figure 5A-C). This might be attributed to the fact that Gef alone has a low delivery efficiency and did not completely induce apoptosis of tumor cells resulting in acquired drug resistance 32.

The limited penetration of drug delivery systems into deep tumor tissue has been one of the major limitations of nanoplatforms in treating cancer. The resected tumor from the mouse injected with Gef/H-dot+Gen/H-dot was examined by histological analysis to confirm the penetration and distribution of drug/H-dot complexes (Figure 5D). In the tissue section images, strong fluorescent signals are observed even in the deep tumor tissues (white dotted line squares) from both the 800 nm and 700 nm NIR channels under the NIR fluorescence microscope. This result suggests that H-dots can deliver drugs deep into tumor tissues due to their nonsticky property and high permeability 31. Additionally, the enhanced antitumoral efficacy of drug/H-dots is due to their high targetability and increased release of Gef from the Gef/H-dot complex in acidic tumor microenvironments (Figure S7, Supporting Information).

### Histopathological analysis of tumors treated with Gef/H-dot800 and Gen/H-dot700 complexes

**Figure 6:**
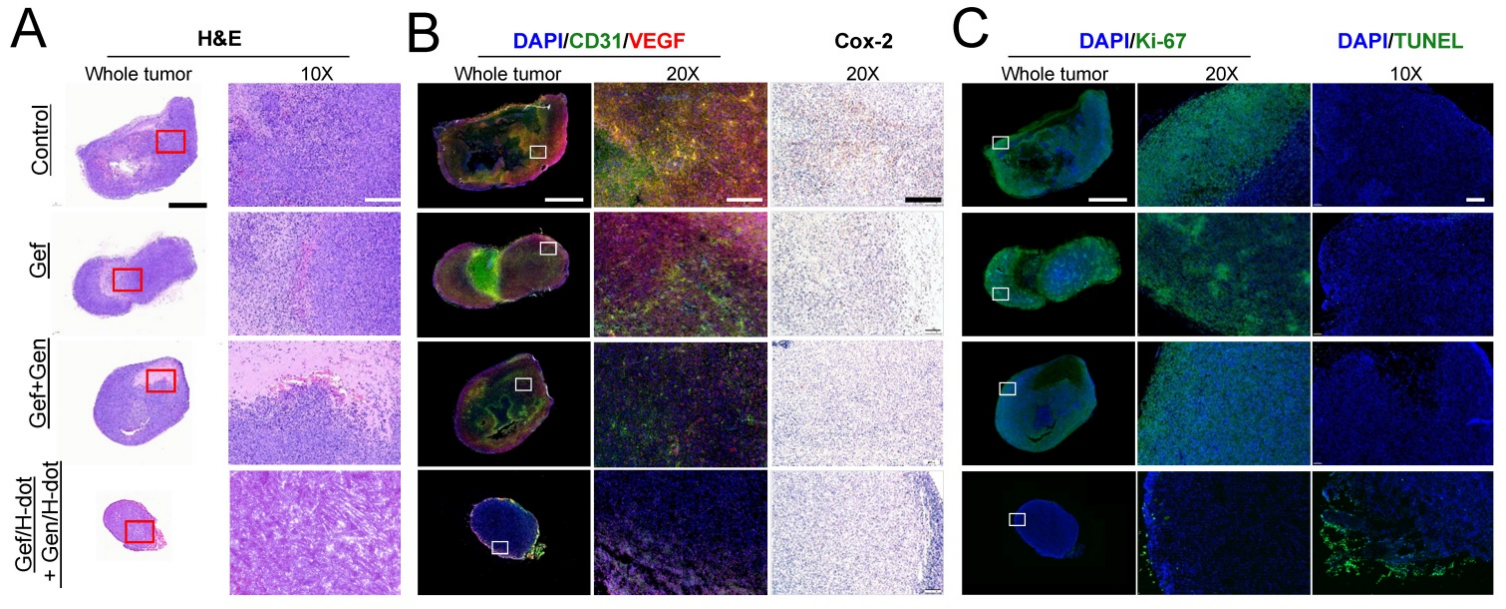
** Histopathological examination of LLC tumors for synergistic therapeutic efficacy of Gef/H-dot+Gen/H-dot group compared to saline, Gef, and Gef+Gen treatment groups. (A)** H&E staining for observation of tumor's histopathological changes. **(B)** CD31 and VEGF staining images for detecting expression and distribution of tumor-associated angiogenesis, and cyclooxygenase-2 (COX-2) staining images for detecting the changes in inflammatory factors in tumor tissues. **(C)** Ki-67 staining and terminal deoxynucleotidyl transferase dUTP nick end labeling (TUNEL) images of resected tumor tissues. Ki-67 staining was used to determine tumor proliferation indexes, and TUNEL was used for observation of tumor cell apoptosis. Scale bar: 2 µm for whole tumor images and 200 µm for the others.

Tumor morphological changes and the possible mechanism were assessed by H&E staining, immunohistochemical analysis, and terminal deoxynucleotidyl transferase (TdT) dUTP nick-end labeling (TUNEL) apoptosis staining as shown in Figure 6. H&E staining showed that high cellularity and no signs of tissue damage were observed, and necrosis was identified at the center area in the saline control group (Figure 6A). In the Gef+Gen and Gef/H-dot+Gen/H-dot groups, the pathology test indicated severe apoptosis and significant tissue loss across a large tumor area, and the necrotic area and cell density were lower compared with Gef and saline control groups. Importantly, the arrangement of LLC tumor cells was sparsest inside the tumor of the Gef/H-dot+Gen/H-dot group. Furthermore, stained tumor tissues from the mice which received Gef, Gef+Gen, and Gef/H-dot+Gen/H-dot showed different potential mechanisms of anti-proliferation and anti-angiogenesis from the saline control group and also showed opposite apoptotic damages compared to the saline group. The immunofluorescence staining of CD31 (green) and VEGF (red) provides clear evidence that the combination treatment of Gef and Gen effectively inhibited angiogenesis compared with the Gef alone treatment. Particularly, the expression of CD31 and VEGF was significantly downregulated in the Gef/H-dot+Gen/H-dot group compared with other groups by changing the tumor microenvironment due to the high efficacy of anti-angiogenesis accompanied with remarkable tumor regression suggesting excellent synergistic therapeutic efficacy (Figure 6B).

Cyclooxygenase-2 (COX-2), an inflammatory mediator, is associated with tumor initiation and progression which contributes to tumorigenesis and differentiation 33. The COX-2/VEGF-dependent pathways can affect tumor-associated angiogenesis, tumor growth, and tumor metastasis 34. VEGF is a key factor in the activation of angiogenic signaling pathways. By targeting VEGF, COX-2/VEGF-dependent pathways could be downregulated to prevent tumor cells from establishing de novo blood vessels 35. The lower expression level of proliferation index Ki-67, inflammation index COX-2, as well as the highest apoptotic levels with TUNEL were detected in the Gef/H-dot+Gen/H-dot treatment group compared with the other three groups (Figure 6C). Taken together, histopathological results suggest that the Gef/H-dot and Gen/H-dot complexes can improve the inhibition of cellular proliferation, angiogenesis, and inflammation and promote apoptosis through multiple molecular pathways in tumor cells and tumor microenvironments compared with the treatment of free drugs.

### Systemic toxicity evaluation of tumor-bearing mice treated with Gef/H-dot and Gen/H-dot complexes

**Figure 7:**
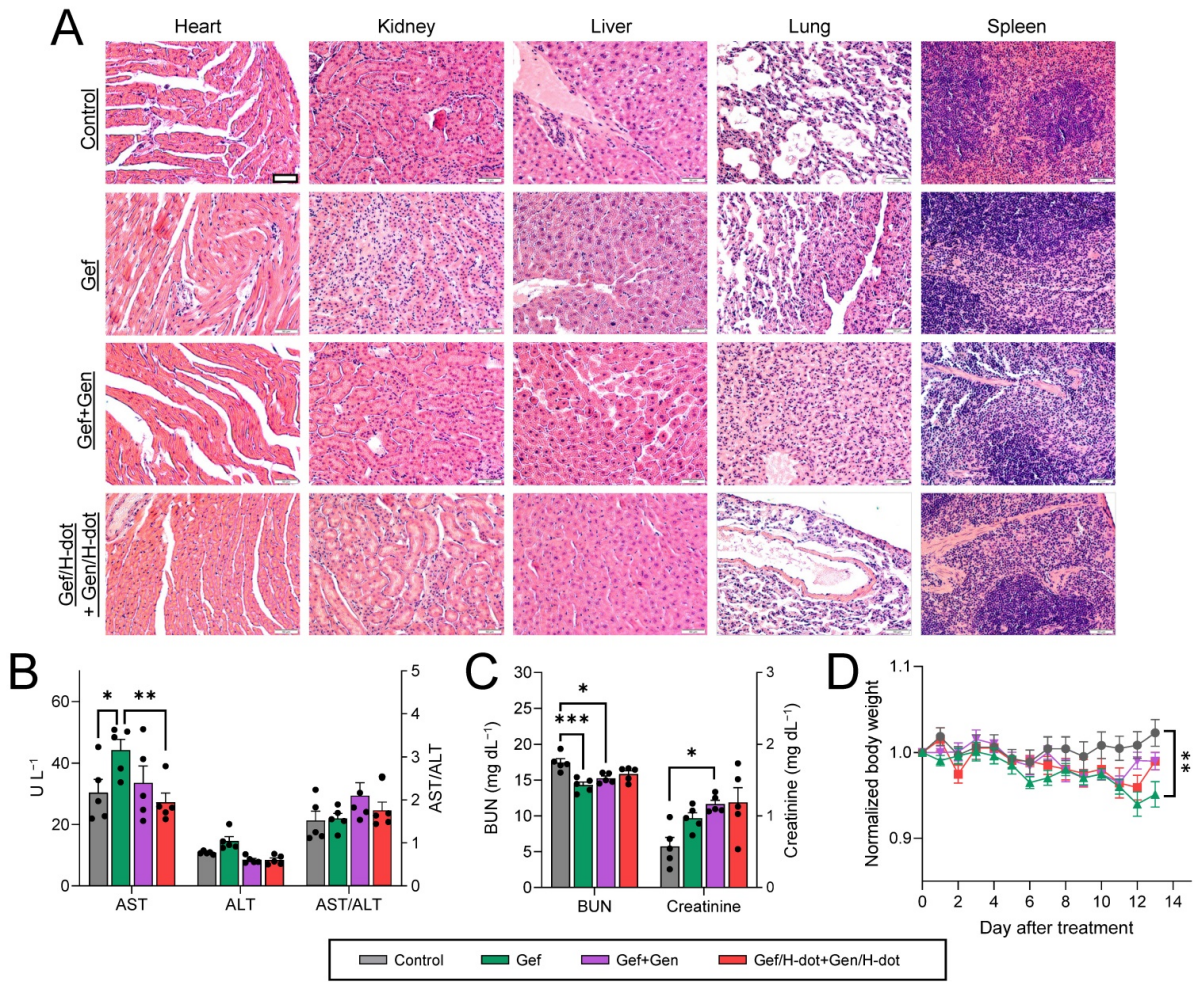
***In vivo* toxicity test in LLC tumor-bearing mice treated with saline, Gef, Gef+Gen, and Gef/H-dot+Gen/H-dot, respectively, for 14 d (*n*= 7 per each group). (A)** H&E staining images (20×) of heart, liver, spleen, lung, and kidney in each treatment group. Scale bar: 200 µm. **(B)** Serum aspartate transferase (AST), alanine transferase (ALT), and AST/ALT ratio. **(C)** Blood urea nitrogen (BUN) and creatinine. **(D)** Body weights of mice for 14 d during the treatments. *p* values < 0.05 were considered significant: *p < 0.05 and **p < 0.01.

During the treatment period of Gef/H-dot+Gen/H-dot, signs and symptoms of toxicity were monitored such as lack of drinking, eating, grooming, and activity, as well as abnormal urination or neurological status, none of which were observed. After sacrificing the mice at D13, the systemic repeated-dose toxicity of Gef/H-dot+Gen/H-dot was further evaluated by histopathological examinations and biochemical analysis. H&E staining of the major organs, such as the heart, kidney, liver, lung, and spleen, showed no apparent tissue damage, inflammation, or morphological changes in the Gef/H-dot+Gen/H-dot treatment group when compared to the saline control group (Figure 7A and Figure S13, Supporting Information). However, pulmonary toxicity such as pulmonary tissue fibrosis and inflammation was observed in the Gef and Gef+Gen treatment groups. This result indicates that Gef/H-dot+Gen/H-dot treatment can significantly reduce the pulmonary toxicity of targeted drugs.

Gef is mostly cleared by hepatic metabolism via cytochrome P450 36, and because of this, some patients experience side effects of hepatotoxicity 37, while Gen has a protective action on adjacent normal healthy organs, which makes this natural isoflavone an attractive key compound for the development of effective drugs for combating malignant disorders 35. In the biochemical analysis results shown in Figure 7B, the AST (aspartate aminotransferase) levels in both Gef+Gen and Gef/H-dot+Gen/H-dot groups showed no significant differences from those in the saline control group; however, these levels were increased in the Gef alone treatment group, indicating liver toxicity (**p < 0.01 compared to Gef/H-dot+Gen/H-dot group). This suggests that H-dots did not induce any additional hepatotoxicity, and that Gen might be protecting the liver cells from Gef-induced hepatotoxicity 38. In addition to the AST result, ALT (alanine transaminase) levels showed a similar trend, and no significant changes in the AST/ALT ratio were observed between all treatment groups.

A low BUN (blood urea nitrogen) level in the Gef group was indicated (**p < 0.001) due to abnormal liver function and weight loss, while the BUN level in the Gef/H-dot+Gen/H-dot group had no significant difference compared to the saline control group (Figure 7C-D). The levels of plasma creatinine in all positive treatment groups increased due to the nephrotoxicity of Gef 39. H-dot did not induce any additional nephrotoxicity; however, it did not appear to significantly reduce the inherent nephrotoxicity of Gef. This indicates that Gef/H-dot+Gen/H-dot complexes did not induce significant renal impairment despite their exclusive renal clearance (Figure 7C). Mouse body weight changes during the treatments were also monitored to assess the systemic toxicity (Figure 7D). The body weights of mice in the Gef group continuously decreased (~5% lost, *p < 0.05 compared to saline) due to low intake and loss of appetite, both side effects of Gef 40, but no other major signs of toxicity were observed 41. In contrast, the mouse bodyweight of the Gef/H-dot+Gen/H-dot complex treatment group had no obvious differences when compared to the saline control group, which can be ascribed to the reduced toxicity of drugs when delivered by H-dots due to their fast renal clearance (Figure 7D).

## Discussion

The mortality rates and survival outcomes of lung cancer remain poor. Thus, the development of an effective theranostic strategy is extremely urgent in the next generation of targeted therapies. Moreover, consequent adverse events have led to a strong desire to explore and search for new targeted therapeutic strategies with fewer side effects. Multifunctional nanoparticles can be used as theranostic nanoplatforms for biomedical imaging, diagnosis, and drug delivery, which show great promise toward therapeutic nanomedicine 42-44. Based on the “design considerations of a modular approach” 31, 45, we have designed and synthesized the renal clearable H-dot as a theranostic nanoplatform. The renal clearable H-dot is an ideal theranostic nanoplatform because it efficiently targets tumors, suppresses tumor growth with drug delivery, and reduces the toxicity of injected drugs by rapid elimination from the body 46. In this work, we demonstrate three distinct features of drug/H-dot complexes to prove the ideality of this theranostic nanoplatform.

First, we performed real-time image-guided surgeries using NIR fluorescent H-dots by delineating the tumor boundary and LNMs with high tumor-to-background ratios. Due to the difficulty of localizing and determining a safe margin for tumors and/or LNMs that may not be visible to the naked eye during surgery without a separate guiding procedure, many surgeons take a great deal of time to work out tumor boundaries and even then are still met with troubles of incomplete dissection or positive surgical margins which result in recurrence and metastases 25, 26. Thus, the usage of intraoperative image-guided surgical procedures for tumor resection, particularly with LNMs, is considered to be the best for the complete removal of neoplastic tissues. In this context, we demonstrated that tumors and LNMs were successfully resected using targeting fluorescent H-dot complexes, which clearly differentiated tumors from the surrounding tissues with high SBRs. In addition, under postoperative fluorescent microscopic examination, tumor lesions can be detected in resected ex vivo specimens which helps pathologists easily and accurately isolate metastatic lymph nodes from the dissected tissues for subsequent pathological testing, as well as make faster judgments on positive excision margins.

The second desirable feature of H-dots is that they offer efficient perioperative chemotherapy for preventing further infiltration and metastases. The combination therapy of Gef and Gen with H-dots significantly downregulated CD31 and VEGF in cancerous regions indicating that the degree of tumor angiogenesis was significantly inhibited, which was accompanied by remarkable tumor regression. Moreover, the COX-2/VEGF-dependent pathway suppresses the function of tumor cells, which affects tumor-associated angiogenesis, tumor growth, and tumor metastasis 34. In this study, the density of LLC tumor cells was lowest inside the tumor with the lowest expression levels of proliferation index Ki-67, inflammation index COX-2, and tumor angiogenesis index VEGF due to their inhibition by the combination of Gef/H-dot and Gen/H-dot. This demonstrates that the Gef/H-dot and Gen/H-dot combination targeted therapy regulates various intracellular targets and signaling pathways simultaneously to slow down tumor progression, which is due to H-dot's ability to take more drugs into tumor sites faster than free drugs alone. In addition, using the dual-channel NIR imaging technique, the delivery of two different classes of drugs (EGFR-TKIs and AIs) was monitored, as well as the therapeutic efficacy. VEGF-EGFR crosstalk is well described; however, drug-drug interactions in Gef/H-dot and Gen/H-dot treatments need further investigation and extensive clinical studies in order to maximize the anti-tumor efficacy and minimize target drug resistance and risk of tumor relapse.

Lastly, the optimal PK profile of target drug/H-dot complexes shows promise in reducing these adverse effects, which could increase treatment adherence in patients. Severe side effects of Gef, including hepatotoxicity, ILD, and severe diarrhea, can be life-threatening and/or impact health-related quality of life 13, 14. The most common grade 3 or 4 adverse effects reported are rash and diarrhea; around 10% of patients discontinue treatment due to adverse effects 37. Fortunately, H-dot drug delivery nanoplatforms modified the pharmacodynamic efficacy and toxicity of free target drugs. The drug/H-dot complexes can evade nonspecific uptake by off-target tissues and be delivered more efficiently to tumor sites owing to their zwitterionic property. In the combination therapy experiment *in vivo*, even the highest dose in the Gef/H-dot+Gen/H-dot treatment group yielded no obvious tissue damage, inflammation, or morphological change of normal organs in both histological and biochemical analyses. Compared with free drugs, combination therapy with drug/H-dot complexes decreased pulmonary inflammation based on the H&E histopathology result and could inhibit pulmonary fibrosis which is a pathological change caused by ILD 47, 48. To the best of our knowledge, our renal clearable nanoplatform is one of the most innovative and promising noninvasive approaches for image guidance and drug delivery applications. This platform has the distinct advantage of dramatically decreasing side effects associated with free drugs, which may be the key to overcoming the barriers to successful clinical translation.

## Conclusion

In summary, we demonstrated that the drug/H-dot complex combination theranostic (Gef/H-dot+Gen/H-dot) is an ideal nanoplatform for efficient tumor targeting and imaging, tumor growth suppression, and reducing the toxicity of free drugs. Drug/H-dot complexes have infinite potential for performing real-time dual-channel NIR fluorescence image-guided staging detection, surgical intervention, and pathological assistance, as well as targeted delivery of anti-tumor drugs (Gef and Gen) simultaneously for a highly effective synergistic combination treatment with less adverse effects. Further investigation will involve exploring the possible mechanism and strategies of H-dot-based drug delivery systems for increasing the accessibility and bioavailability of multiple targeted drugs for various treatments which is of great clinical interest.

## Materials and Methods

### Materials

Genistein was purchased from Biotang Inc. Cell Counting Kit-8 (CCK-8) was purchased from Dojindo Molecular Technologies (USA). Gefitinib salt and biochemical assay reagents kits (for aspartate aminotransferase, alanine aminotransferase, and serum creatinine) were purchased from Cayman Chemical (USA). The QuantiChrom™ urea assay kit was purchased from Bioassay Systems (USA). The LLC cells were purchased from American Type Culture Collection (MD, USA). Dimethyl sulfoxide (DMSO), Bovine serum albumin (BSA), sodium hydroxide (NaOH), HCl (12.1 M) were purchased from MilliporeSigma (USA) and used without further purification.

### Preparation of Gef/H-dot and Gen/H-dot inclusion complexes

To prepare the Gef/H-dot800 complex, Gef salt was dissolved in DI water (2 mM). H-dot800 powder was dissolved in DI water (1 mM), and the pH was adjusted to 5.33 using HCl solution (~0.3 M). Afterward, the above solutions were mixed at a volume ratio of 1:1 and vortexed for 60 min at room temperature. Then, the solution was centrifuged at 14,000 rcf for 10 min to precipitate impurities and obtain the product complex from the supernatant, which was freeze-dried after collection. The molar ratio of Gef to H-dot was determined using UV spectrophotometry. To prepare the Gen/H-dot700 complex, Gen was dissolved in DMSO (100 mM). H-dot powder was dissolved in DI water (2 mM). The Gen solution was added slowly to the H-dot solution at a volume ratio of 1:9 (Gen:H-dot) with continuous shaking. Afterward, the mixture was vortexed for 4 h at room temperature and stored at 4 ºC overnight. To collect the product, the solution was centrifuged at 14,000 rcf for 10 min to precipitate impurities, and the supernatant was purified using gel filtration chromatography (GFC) and then lyophilized. The mole ratio of Gen to H-dot in the complex was determined using UV spectrophotometry.

### CDOCKER protocol in Discovery Studio 3.0 software

The 3D structure of β-CD was selected as the docking receptor (Protein Data Bank ID code: 1BFN). The ligand was energy-minimized with the CHARMm force-field, and the model was selected with the lowest binding energy using the CDOCKER protocol in Discovery Studio 3.0 software. The pictures in Figure 1B were edited with PyMOL 2.3.1 Visualizer.

### pH-responsive drug release tests of Gef/H-dot and Gen/H-dot complexes

The pH-responsive drug release tests of Gef/H-dot and Gen/H-dot complexes were done using rapid equilibrium dialysis devices (8 kDa MWCO, Thermo Scientific). Gef/H-dot and Gen/H-dot complexes were separately dissolved in PBS solutions of pH 6.0 and 7.4, respectively, at a concentration of 500 μM with respect to H-dot. For each complex, sample chambers were filled with 200 μL of complex solution, and corresponding buffer chambers were filled with 400 μL of PBS solution at a pH equivalent to that of the complex solution. The plate was put on an up-and-down shaker at 20 rpm at 37 ºC. 200 μL of the release solutions were pipetted from the buffer chambers at each sampling time; 0.25, 0.5, 1, 1.5, 2, 4, 6, 8, and 24 h, and 200 μL of fresh PBS of respective pH. The concentration of the Gef and Gen were calculated by measuring UV absorbances at 332 nm and 258 nm, respectively. The accumulated drug release percentage (*Q_n_*) was calculated by the following equation:



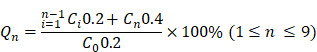



, where *C_i_* and *C_n_* are the concentrations of the drug in the buffer chamber at each time point, and *C_0_* is the initial concentration of the drug in the sample.

### In vitro therapeutic efficacy test of drug/H-dot complexes

LLC cells were incubated in DMEM (Mediatech, Herndon, VA) supplemented with 10% FBS and 1% penicillin/streptomycin in a humidified incubator at 37 ºC before experiments. For the *in vitro* efficacy test, cells were seeded at a density of 3 × 10^3^ cells/well in 96-well plates. The plates were incubated for 24 h before treatment with H-dot, Gen, Gef, Gef/H-dot, and Gen/H-dot ranging from 0.001 to 50 μM for 24 h. To confirm the combination therapeutic efficacy, additional plates were treated with H-dot, Gef, Gen, Gef+Gen, and Gef/H-dot+Gen/H-dot for 24 h and 48 h. The cells without any treatments were used as a control. The therapeutic efficacy of each drug was evaluated by the CCK-8 assay and microscopy. For the CCK-8 assay,10 µL of CCK-8 solution was added to each well in the plates with an incubation time of 2 h, and then the absorbance was measured at 450 nm using a microplate reader (SpectraMax, Molecular device). All experiments were carried out with five replicates. The survival rate was calculated according to the equation below:



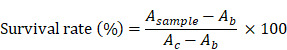



, where *A_sample_*, *A_b_*, and *A_c_* are absorption values from drug treatments, blank, and negative control, respectively.

The mean EC50 value was calculated by the following equation:



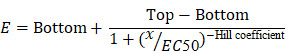



, where E is the % cell viability, Top and Bottom are plateaus in the % cell viability, *x* is the logarithm of the concentration, and the Hill coefficient reflects the slope of the curve.

### In vivo biodistribution and pharmacokinetics of drug/H-dot complexes

Animals were housed in an AAALAC-certified facility and were studied under the supervision of MGH IACUC in accordance with the approved institutional protocol (2016N000136). Prior to injection of treatments, six-week-old CD-1 mice (male; 25-30 g from Charles River Laboratories (Wilmington, MA) were anesthetized with isoflurane & oxygen, and blood was sampled in capillary tubes (Fisher Scientific, Pittsburgh, PA) at the time point 0 min by slightly cutting the end of the tail. H-dot, Gef/H-dot, and Gen/H-dot in saline were intravenously injected at the same dose level as the imaging experiments. Blood samples were obtained at 1, 3, 5, 10, 30, 60, 120, 180, and 240 min post-injection, and the fluorescence intensities of serum samples in capillary tubes were measured to calculate distribution (*t*_1/2α_) and elimination (*t*_1/2β_) half-life values (*n* = 3-4 for each group). After 4 h post-injection, mice were sacrificed to image organs (liver, lung, spleen, kidney, stomach, brain, intestine, and bladder). Results were presented as a bi-exponential decay curve using GraphPad Prism software version 9.0 (GraphPad, San Diego, CA).

### In vivo real-time targeting and image-guided surgery

To establish the LLC tumor model, C57BL/6 mice (Taconic Farms, Germantown, NY) were subcutaneously injected with 2×10^5^ LLC cells suspended in 100 µL of DMEM/Matrigel (50 *v/v*%) in the flank region. For the orthotopic lung cancer model, LLC cells (2×10^6^) suspended in 0.1 mL of saline were injected into the lung of the mice from the trachea. Real-time imaging was performed when the tumors were formed after two weeks. LLC lung tumor-bearing mice were intravenously injected with Gef/H-dot800 and Gen/H-dot700.* In vivo* fluorescence imaging was performed using our NIR imaging system (K-FLARE) on two channels, 800 nm and 700 nm (0, 1, 2, 4, and 24 h post-injection). At 24 h post-injection, the mice were sacrificed for ex vivo imaging and histological evaluations (heart, liver, stomach, lung, kidney, brain, spleen, intestine, bladder, pancreas, bone, muscle, and tumor).

### In vivo anti-tumor efficacy test

A subcutaneous LLC tumor model was established in C57BL/6 mice (Taconic Farms, Germantown, NY) by injecting 2 × 10^5^ LLC cells into the flank region which can be considered the subcutaneous metastasis in NSCLC patients 49, 50. The mice were divided into four treatment groups (n = 4) when the mean tumor volume reached approximately 30 mm^3^, designated as D0. Each group received different treatments: saline, 25 mg/kg Gef (dissolved in 25 *w/v*% cremophor-saline solution), 25 mg/kg Gef + 25 mg/kg Gen (dissolved in 31 *w/v*% cremophor-saline solution), and 413 mg/kg Gef/H-dot + 764 mg/kg Gen/H-dot, given every day 11 times within the 14-day study period. Gef/H-dot+Gen/H-dot in saline was injected through the tail vein while all other treatments were given intraperitoneally. After injection, *in vivo* fluorescence imaging was performed using our NIR imaging system with two channels, 800 nm and 700 nm. Mice were sacrificed at D14 for ex vivo imaging and histological evaluations. Tumor volume and body weight were measured every day during the treatment. The length (L) and width (W) of the tumors were measured with a digital caliper, and the volume (V) was calculated by the equation V = ½LW^2^. The tumors were excised, and the weight was measured.

### Tissue histopathology evaluation

Heart, liver, spleen, lung, kidney, and tumor tissues were fixed in 10% neutral buffered formalin for more than 8 h. Then, the tissues were dehydrated in ethanol, embedded in paraffin, and sectioned into slices (5 µm). After rinsing with PBS, the fixed sections were counterstained with nuclear fast red, dehydrated by ethanol, transferred into xylene, and finally mounted according to the standard protocol. Then, those sections were stained with hematoxylin and eosin (H&E) for pathology observation under optical microscopy. Apoptosis of the tumors was determined by TUNEL. For analysis of cell proliferation, sections were incubated with an anti-Ki67 antibody. As to the analysis of inflammatory responses at tumor sites, sections were incubated with anti-COX-2 antibody. COX-2 staining with brown color was carried out using the glucose oxidase-diaminobenzidine (DAB) method. The expression of tumor angiogenesis-related factors VEGF and vascular endothelial marker CD31 were evaluated by staining with their respective antibodies.

### Toxicity study

To evaluate the potential long-term toxicity, blood samples were obtained by cardiac puncture at the end of the anti-tumor efficacy test (D14). These blood samples were stored without anticoagulant and then centrifuged at 5,000 rpm for 5 min. Serum was stored at -80ºC until further assays. The biochemical parameters tested were a liver function panel (aspartate aminotransferase (AST) and alanine aminotransferase (ALT)) and kidney function indication (blood urea nitrogen (BUN) and creatinine (CREA)). All parameters were tested using commercially available assay kits, and the absorbance was immediately measured by a plate reader.

### Statistical analysis

The fluorescence and background intensities of a region of interest over each tissue were quantified using customized imaging software and ImageJ v1.48 (National Institutes of Health, Bethesda, MD). The signal-to-background ratio (SBR) was calculated as SBR = fluorescence/background, where the background is the fluorescence intensity of muscle. Data are reported as mean ± s.e.m. with a minimum of three biological replicates. The student's *t*-test statistical analysis was performed to evaluate the significance of the experimental data. The differences among groups were determined using one-way ANOVA analysis to assess the statistical differences among more than two groups. A *p* value of less than 0.05 was considered significant. The data was indicated with *p < 0.05, **p < 0.01, ***p < 0.001, and ****p < 0.0001.
