# Supplementary material for: Image-guided drug delivery of nanotheranostics for targeted lung cancer therapy
Source: Theranostics. 2022 May 13;12(9):4147–62. doi: 10.7150/thno.72803 (PMC9169367; doi:10.7150/thno.72803)
Supplement: Supplementary file 1 — Supplementary methods, tables, figures, and movie legend. [file thnov12p4147s1.pdf]

Supplementary Information

Image-Guided Drug Delivery of Nanotheranostics for Targeted Lung Cancer Therapy

Xiaoran Yin,[1,2,#] Yanan Cui,[2,3,#] Richard S. Kim,[2] Wesley R. Stiles,[2]
Seung Hun Park[2], Haoran Wang,[2] Li Ma,[4] Lin Chen,[5] Yoonji Baek,[2] Satoshi Kashiwagi,[2]
Kai Bao,[2] Amy Ulumben,[2] Takeshi Fukuda,[2] Homan Kang,[2,*] and Hak Soo Choi[2,*]

[1]Department of Oncology, The Second Affiliate Hospital of Xi'an Jiaotong University, Xi'an, Shaanxi, 710004, China
[2]Gordon Center for Medical Imaging, Department of Radiology, Massachusetts General Hospital and Harvard Medical School, Boston, MA 02114, USA
[3]School of Pharmacy, Jining Medical College, Rizhao, Shandong, 276826, China
[4]Department of Pathology, The Second Affiliate Hospital of Xi'an Jiaotong University, Xi'an, Shaanxi, 710004, China
[5]Department of Pathology, Shaanxi Province People's Hospital, Xi'an, Shaanxi, 710068, China

#These authors contributed equally to this work.

*Correspondence to HK at hkang7@mgh.harvard.edu or HSC at hchoi12@mgh.harvard.edu

This file includes:

Supplementary Methods
Table S1. In vitro dose information
Table S2. Pharmacokinetic parameters of Gef/H-dot and Gen/H-dot
Table S3. In vivo dose information
Figure S1. SEC-HPLC result of ZW800-H-dot
Figure S2. Protein standard curve for hydrodynamic diameter determination
Figure S3. Ninhydrin test of ZW800-CDPL$^{\pm}$ (H-dot)
Figure S4. ^{1}H-NMR spectra of β-Cyclodextrin, EPL$^+$, and CDPL$^+$ in D_2O
Figure S5. Chemical structure of ZW800-1C and ZW700-1C
Figure S6. Determination of the molar ratio of drug and H-dot
Figure S7. Drug release test of drug/H-dots
Figure S8. In vitro therapeutic efficacy test
Figure S9. Biodistribution of H-dot, Gef/H-dot, and Gen/H-dot
Figure S10. Pharmacokinetics of H-dot
Figure S11. Tumor targeting and delivery efficiency of Gef/H-dot+Gen/H-dot.
Figure S12. Image-guidance tumor pathology test
Figure S13. Histological assessment of toxicity on major organs treated with drugs
Movie S1. Real-time movie of LLC lung tumor-bearing mice injected with Gef/H-dot at 24 h post-injection.

SUPPLEMENTARY METHODS

Reagents and materials.

Epsilon-poly-L-lysine (ε-poly-L-lysine, EPL; MW ~3,900) was purchased from BOC Sciences (Shirly, NY). Beta-cyclodextrin (β-CD; MW ~1,134) was purchased from Tokyo Chemical Industry (Tokyo, Japan). Acetic acid, ethyl acetate (EA), Dess-Martin periodinane (DMP), ninhydrin, succinic anhydride, deuterium oxide (D_2O), anhydrous dimethyl sulfoxide (DMSO), sodium acetate, sodium borohydride, and sodium hydroxide were purchased from Sigma-Aldrich (Saint Louis, MO). Ninhydrin agent and succinic anhydride (SA) were purchased from Acros Organics (Morris Plains, NJ). Acetone was purchased from Fisher Scientific (Pittsburgh, PA).

Preparation of H-dots.

Conversion of β-CD to mono-aldehyde β-CD: To prepare mono-aldehyde β-CD (Ald-CD), 15 g of β-CD (13.22 mmol) was dissolved in anhydrous DMSO (100 mL) in a round bottom flask equipped with a stir bar. To this solution was added 6.73 g of DMP (15.86 mmol), and after dissolving, the solution was stirred at room temperature for 2 h. After 2 h, the solution was precipitated in 750 mL of cold (4 ºC) EA/acetone (20% v/v) and stored at 4 ºC overnight. The precipitate was retrieved by vacuum filtration and dissolved again in a minimal amount of DMSO. This solution was then poured into 750 mL of cold EA/acetone (20% v/v) and centrifuged at 3000 rpm for 15 min. The supernatant was discarded, and the dissolution of Ald-CD in deionized water (DIW) and precipitation in EA/acetone was repeated two more times for a total of three precipitation steps to remove soluble impurities. After the last precipitation, the precipitate was dissolved in 75 mL of DIW and stirred for 1 h followed by lyophilization to remove complexed acetone and DMSO. After lyophilization, a white solid (16 g) was recovered as the product (106.6% due to unremoved DMSO/Acetone).

Conjugation of Ald-CD onto EPL: 16 g of Ald-CD (14.10 mmol) was dissolved in 550 mL of acetate buffer (0.2 M, pH 4.5) followed by the addition of 2.2 g of EPL (0.564 mmol). After stirring for 2 h, 1 g of sodium borohydride (26.45 mmol) was added into the reaction mixture to reduce the Schiff base to a secondary amine. The mixture was stirred for an additional 72 h at room temperature. After this time period, dynamic dialysis was carried out in a cellulose membrane with a molecular weight cutoff (MWCO) of 6-8 kDa for 24 h against DIW. The dialyzed solution was frozen at -80 ºC and lyophilized to retrieve 3.75 g of an off-white fluffy

solid. To characterize the β-CD-conjugated EPL (CDPL), the number of β-CD grafted to EPL was determined using ^{1}H-NMR spectroscopy (Figure S4). Additionally, size exclusion chromatography (SEC) was conducted to determine the purity of the CDPL and to confirm the absence of un-grafted Ald-CD (Figure S1).

Conjugation of NIR fluorophore onto CDPL: A solution of ZW800-1C-NHS ester was prepared by dissolving 22.9 mg of the NIR fluorophore (22.38 μmol) in 1 mL of DMSO [1]. A CDPL solution was prepared in a round bottom flask equipped with a stir bar by dissolving 250 mg of CDPL (18.65 μmol, MW 13,403) in 25 mL of PBS (pH 9.0). With vigorous stirring, the ZW800-1C-NHS ester solution was added dropwise into the CDPL solution. The reaction mixture was stirred for 3 h, and the pH was maintained at 9.0 throughout by adding 0.6 M aqueous sodium hydroxide as needed. After 3 h, the product was precipitated in 250 mL of EA/acetone (20% v/v) and centrifuged at 3000 rpm for 15 min. The supernatant was discarded, and the dissolution of ZW800-CDPL in DIW and precipitation in EA/acetone was repeated two more times. The resulting product was dried in vacuo overnight to yield a green solid. For ZW700-1C conjugated CDPL, ZW700-1C-NHS ester (17.4 mg, 18.1 μmol) was used instead of ZW800-1C-NHS ester and the steps above were repeated to retrieve a blue solid [2].

Succinylation of ZW700- or ZW800-CDPL: To obtain zwitterionic H-dots, 152.8 mg of succinic anhydride (1.53 mmol) in DMSO (250 mg/mL) was added to a solution of 1:9 dye-CDPL: CDPL by mass (1.8 g, 130 μmol) in PBS (pH 9.0; 10 mg/mL), and the mixture was stirred for 30 min at room temperature. The pH was maintained at 9.0 throughout the reaction by adding 0.6 M sodium hydroxide as needed. Once the partial succinylation was confirmed by the ninhydrin test, the product was dialyzed in a cellulose membrane with a molecular weight cutoff (MWCO) of 6~8 kDa for 24 h against DIW. The resulting solution was frozen at -80 ºC and lyophilized to afford the final zwitterionic NIR fluorophore-CDPL$^{\pm}$, also known as H-dot [3, 4].

Characterization of H-dots.

Ninhydrin test for estimation of the number of amine groups: Ninhydrin reagent was prepared by dissolving 0.8 g of ninhydrin in 10 mL of ethanol (8 *wt*%). 1 mL of the reagent solution was pipetted into three separate 10 mL scintillation vials. To one vial was added 20 μL of the reaction mixture that was taken before the addition of SA as a reference, to another vial was added 20 μL of the reaction mixture at 30 min after the addition of SA, and to the last vial

was added 20 μL of PBS as a negative control. The vials were vortexed, placed in a boiling water bath for 5 min, and then placed in an ice bath for 3 min to cool. The absorbance was measured at 570 nm of each diluted solution using a UV/Vis/NIR spectrometer (USB2000, Ocean Insight, Dunedin, FL), employing the negative control as a blank. The absorbance at 570 nm of the reaction mixture at 30 min was roughly half that of the reference mixture, indicating that approximately half of all free amines had been succinylated (Figure S3), imparting a zwitterionic surface to the H-dot.

Size-exclusion chromatography analysis: The purity of H-dot was measured using size-exclusion chromatography (SEC) on the Waters HPLC system consisting of a Waters e2695 separations module and Waters 2998 PDA detector. The column used was a BioResolve SEC mAb 200Å, 2.5 μm 7.8x300 mm column equipped with a BioResolve SEC mAb 4.6x30 mm guard column. The mobile phase was isocratic with 10 mM PBS for 30 min at a flow rate of 0.575 mL/min. Each component in the reaction mixture could be identified by its retention time (R_t) and absorbance wavelength.

Hydrodynamic diameter analysis: To measure the hydrodynamic diameter (HD) of H-dot, SEC was performed as described above to obtain the R_t of the H-dot sample. A calibration standard curve of known HDs was obtained by injecting 10 μL of a protein standard mixture (BEH125 and BEH200 SEC Protein Standard Mix; Waters) containing aprotinin (6.5 kDa, 1.96 nm), ribonuclease (13.7 kDa, 3.28 nm), myoglobin (16.7 kDa, 4.1 nm), ovalbumin (44 kDa, 6.10 nm), bovine serum albumin (66.4 kDa, 7.8 nm), immunoglobulin G (150 kDa, 10.6 nm) and thyroglobulin (660 kDa, 17 nm) to the HPLC with the same mobile phase and flow rate described above. Uracil (112 Da) was included in the protein mixture but excluded from the calibration curve as it falls outside of the target HD range. The partition coefficient (K_{av}) was obtained for each protein by the following equation: $K_{av}=(V_e-V_0)/(V_c-V_0)$, where V_0, V_c, and V_e are column void volume, geometric column volume, and eluent volume, respectively. The K_{av} of each protein was plotted against the logarithm of its HD, and a sigmoid curve was fit to the data. To find the HD of an unknown sample, the sample's R_t is converted to K_{av} which can be plugged into the sigmoid curve equation to solve for HD (Figure S2).

^{1}H-NMR analysis: To determine the β-CD conjugation ratio on the EPL chain, 5 mg of CDPL was dissolved in 600 μL of D_2O in a 1.5 mL microtube. After complete dissolution, the solution

was transferred to a clean NMR tube. ^{1}H-NMR spectroscopy was performed on the sample, and the following equation was used to calculate the conjugation ratio:

$$Conjugation\ Ratio = \frac{64 \times H1}{7 \times \varepsilon}$$

, where H1 is the integral area of H1 on β-CD positioned at δ5.06 ppm, and ε is the integral area of the ε protons on EPL positioned at δ3.20 ppm (Figure S4).

References

1. Hyun H, Owens EA, Narayana L, Wada H, Gravier J, Bao K, et al. Central C-C bonding increases optical and chemical stability of NIR fluorophores. RSC Adv. 2014; 4: 58762-8.

2. Hyun H, Henary M, Gao T, Narayana L, Owens EA, Lee JH, et al. 700-nm zwitterionic near-infrared fluorophores for dual-channel image-guided surgery. Mol Imaging Biol. 2016; 18: 52-61.

3. Kang H, Gravier J, Bao K, Wada H, Lee JH, Baek Y, et al. Renal Clearable Organic Nanocarriers for Bioimaging and Drug Delivery. Adv Mater. 2016; 28: 8162-8.

4. Kang H, Stiles WR, Baek Y, Nomura S, Bao K, Hu S, et al. Renal Clearable Theranostic Nanoplatforms for Gastrointestinal Stromal Tumors. Adv Mater. 2020; 32: e1905899.

Supplementary Tables and Figures

Table S1. *In vitro* dose information

**Dose**	**Gef**	**Gen**	**H-dot800**	**H-dot700**	**Gef/H-dot800+Gen/H-dot700**			
					**Gef/H-dot800**		**Gen/H-dot700**	
					H-dot based	Gef based	H-dot based	Gen based
**μM**	40	30	40	30	30	60	40	80

Table S2. Pharmacokinetic parameters of Gef/H-dot and Gen/H-dot (n = 4 per group, mean ± s.e.m.)

**Pharmacokinetic parameter**	**Gef/H-dot800**	**Gen/H-dot700**
Molecular weight ($g \cdot mol^{-1}$)	~16,370	~16,120
Injected dose ($nmol \cdot kg^{-1}$)	2,500	2,500
$t_{½α}$ (min)	9.08 ± 10.37	5.85 ± 2.01
$t_{½β}$ (min)	23.87 ± 4.09	28.72 ± 5.74
Area under the curve (AUC, $min \cdot nmol \cdot mL^{-1}$)	475.0 ± 128.86	499.9 ± 81.64
Plasma clearance (CL, $mL \cdot min^{-1}$)	0.124 ± 0.02	0.124 ± 0.02
Volume of distribution (V_d, $mL \cdot kg^{-1}$)	186.84 ± 29.35	207.08 ± 20.68

Table S3. *In vivo* dose information

**Dose per day**	**Gef**	**Gef+Gen**		**Gef/H-dot800**		**Gef/H-dot800+Gen/H-dot700**			
						**Gef/H-dot800**		**Gen/H-dot700**	
		Gef	Gen	H-dot based	Gef based	H-dot based	Gef based	H-dot based	Gen based
**mg kg^{-1}**	25	25	25	388	25	388	25	739	25
**μmol kg^{-1}**	51.7	51.7	92.6	25.8	51.7	25.8	51.7	49.3	92.6

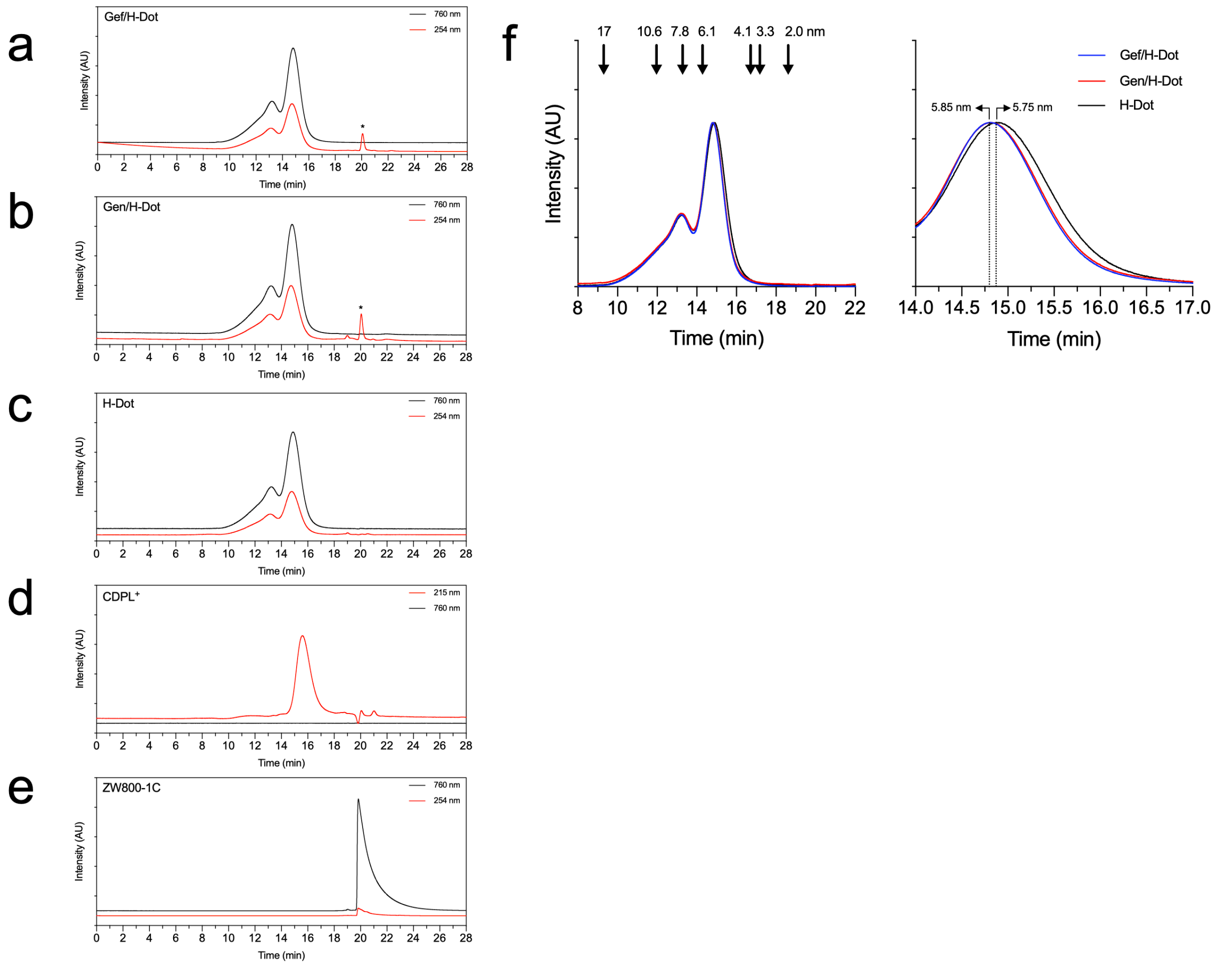

Figure S1. SEC-HPLC result of Gef/H-Dot, Gen/H-Dot, H-Dot, CDPL$^+$, ZW800-1C. SEC-HPLC was employed to confirm the purity of the product, quality of the reaction, and hydrodynamic size. The analyses were conducted on a Waters BioResolve SEC mAb 200Å, 2.5 µm 7.8x300 mm column with an isocratic elution of 10 mM PBS at a flow rate of 0.575 ml/min. (A) Drug complex Gef/H-Dot, with a retention time of 14.806 min in the 760 nm channel. (B) Drug complex Gen/H-Dot, with a retention time of 14.802 min in the 760 nm channel. A small amount of uncomplexed drug is observed in the 254 nm channel around 20 minutes in A and B, indicated with an asterisk. (C) H-dot, with a retention time of 14.883 min in the 760 nm channel. (D) The intermediate product, β-CDPL, with a retention time of 15.606 min in the 215 nm channel, no signals detected in the 760 nm channel. (E) Free ZW800-1C, with a retention time of 19.833 min in the 760 nm channel. (**F**) Comparison of Gef/H-Dot, Gen/H-Dot, and H-Dot SEC profiles, arrows and numbers indicate the retention time maxima and corresponding HD of standard proteins: Aprotinin, 2.0 nm; ribonuclease, 3.3 nm; myoglobin, 4.1 nm; ovalbumin, 6.1 nm; bovine serum albumin (BSA), 7.8 nm; immunoglobulin G (IgG), 10.6 nm; ferritin, 12.2 nm

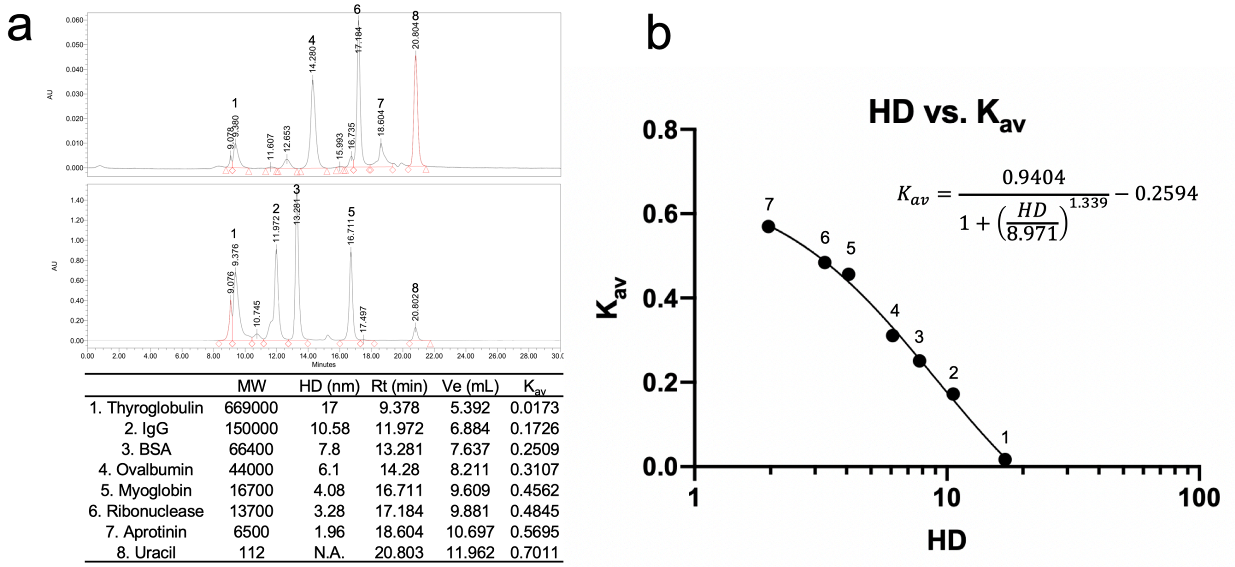

	MW	HD (nm)	Rt (min)	Ve (mL)	K_{av}
1. Thyroglobulin	669000	17	9.378	5.392	0.0173
2. IgG	150000	10.58	11.972	6.884	0.1726
3. BSA	66400	7.8	13.281	7.637	0.2509
4. Ovalbumin	44000	6.1	14.28	8.211	0.3107
5. Myoglobin	16700	4.08	16.711	9.609	0.4562
6. Ribonuclease	13700	3.28	17.184	9.881	0.4845
7. Aprotinin	6500	1.96	18.604	10.697	0.5695
8. Uracil	112	N.A.	20.803	11.962	0.7011

Figure S2. Protein standard curve for hydrodynamic diameter determination. (A) Elution chromatogram of protein standard mix through a Waters BioResolve SEC mAb 200Å, 2.5 µm 7.8x300 mm column monitored at 215 nm with table indexing each component of the protein mix. (B) Semi-log plot relating the partition coefficient (K_{av}) to hydrodynamic diameter (HD, nm) of each component in the protein mixture, fitted with a sigmoid curve. Uracil (8) is excluded from the standard curve as it falls below the lower molecular weight limit of the SEC column.

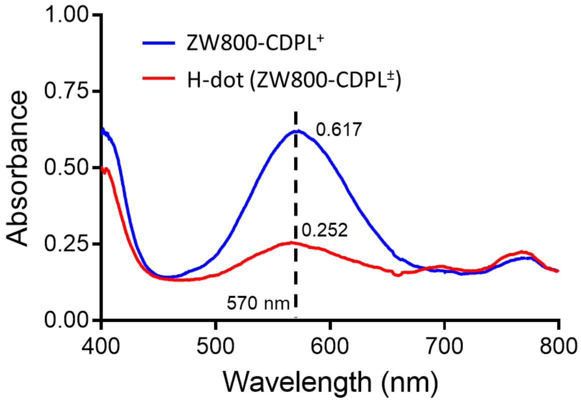

Figure S3. Ninhydrin test of ZW800-CDPL$^{\pm}$ (H-dot). The ninhydrin test was employed to confirm the succinylation ratio of the product molecule. To achieve the zwitterionic property of H-dot, the anionic molecule (succinate) should replace at least 50% of the cationic primary amines on CDPL+. Taking the absorbance at 570 nm of the ninhydrin test solution, the ratio of free primary amines on the raw molecule (ZW800-CDPL^{+}) to free primary amines on the final product (ZW800-H-dot) was determined.

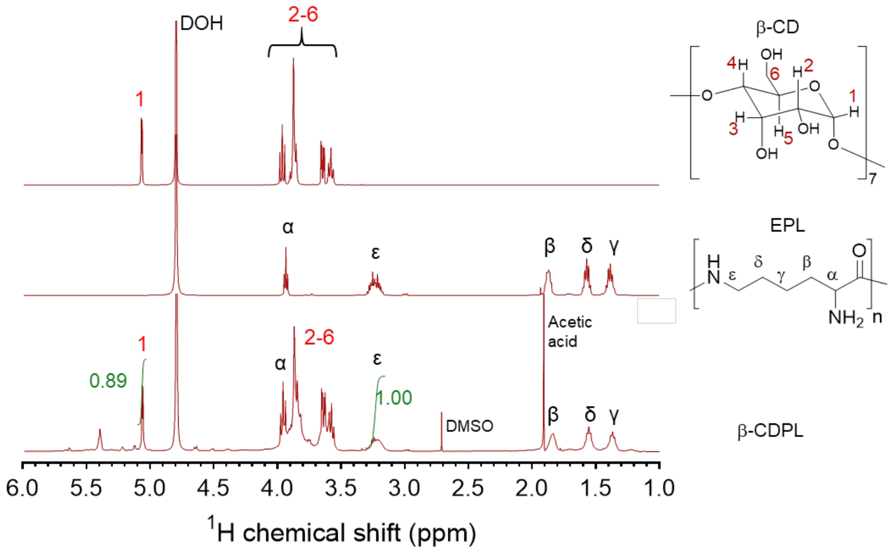

Figure S4. ¹H-NMR spectra of β-Cyclodextrin, EPL$^+$, and CDPL$^+$ in D_2O. The conjugation ratio of β-CD on EPL$^+$ was confirmed by comparing NMR spectra of starting materials and the final product. The H1 peak from β-CD and the ε peak from EPL$^+$ were integrated to calculate the β-CD conjugation ratio per EPL$^+$.

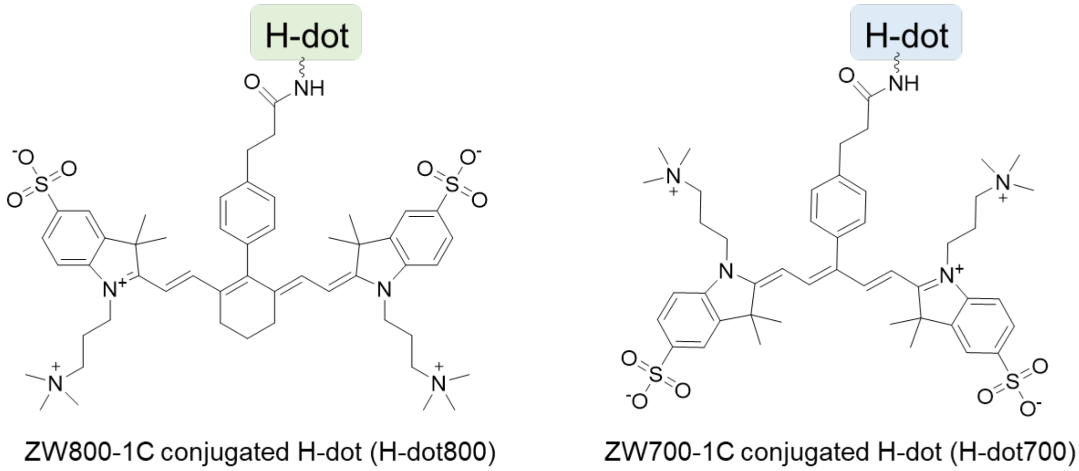

Figure S5. Structure of ZW800-1C and ZW700-1C which were conjugated to H-dots.

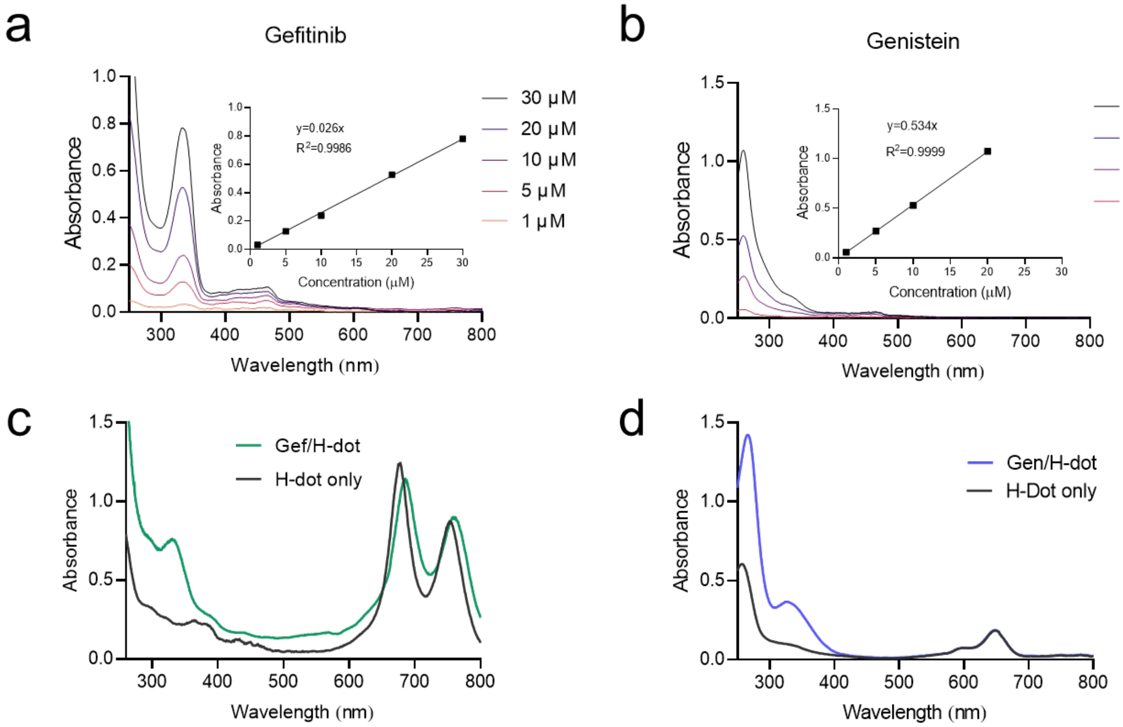

e

	Molar ratio	%EE	%LC
Gef/H-Dot700	1.8	90	4.9
Gen/H-Dot800	2.0	36	3.4

Figure S6. **Determination of the molar ratio of drug to H-dot.** (A) UV absorption spectra of standard solutions of Gef and (B) Gen. Standard curve fittings of Gef and Gen with a linear regression of concentration vs. absorbance (insets). (C) UV absorption spectra of H-dot (black line) and Gef/H-dot (blue line) (D) UV absorption spectra of H-dot (black line) and Gen/H-dot (green line). (E) Molar ratios, encapsulation efficiency (%EE), and loading capacity (%LC) of Gef/H-Dot and Gen/H-Dot. Molar ratio calculated as (moles of drug)/(moles of nanoparticle). Encapsulation efficiency calculated as (mass of encapsulated drug)/(total mass of drug added)×100. Loading capacity calculated as (mass of encapsulated drug)/(total mass of nanoparticles)×100.

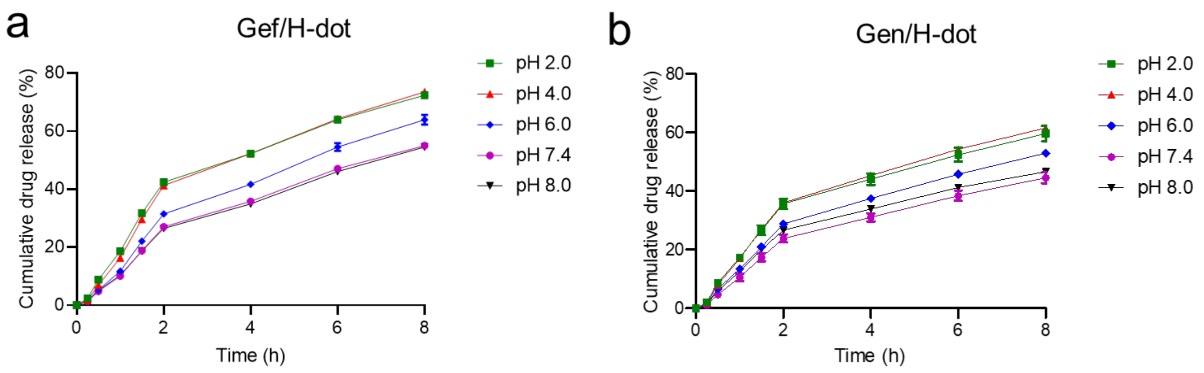

Figure S7. pH-dependent drug release test. (A) Cumulative drug release percentage vs. time at different pH values for Gef/H-dot and (B) Gen/H-dot.

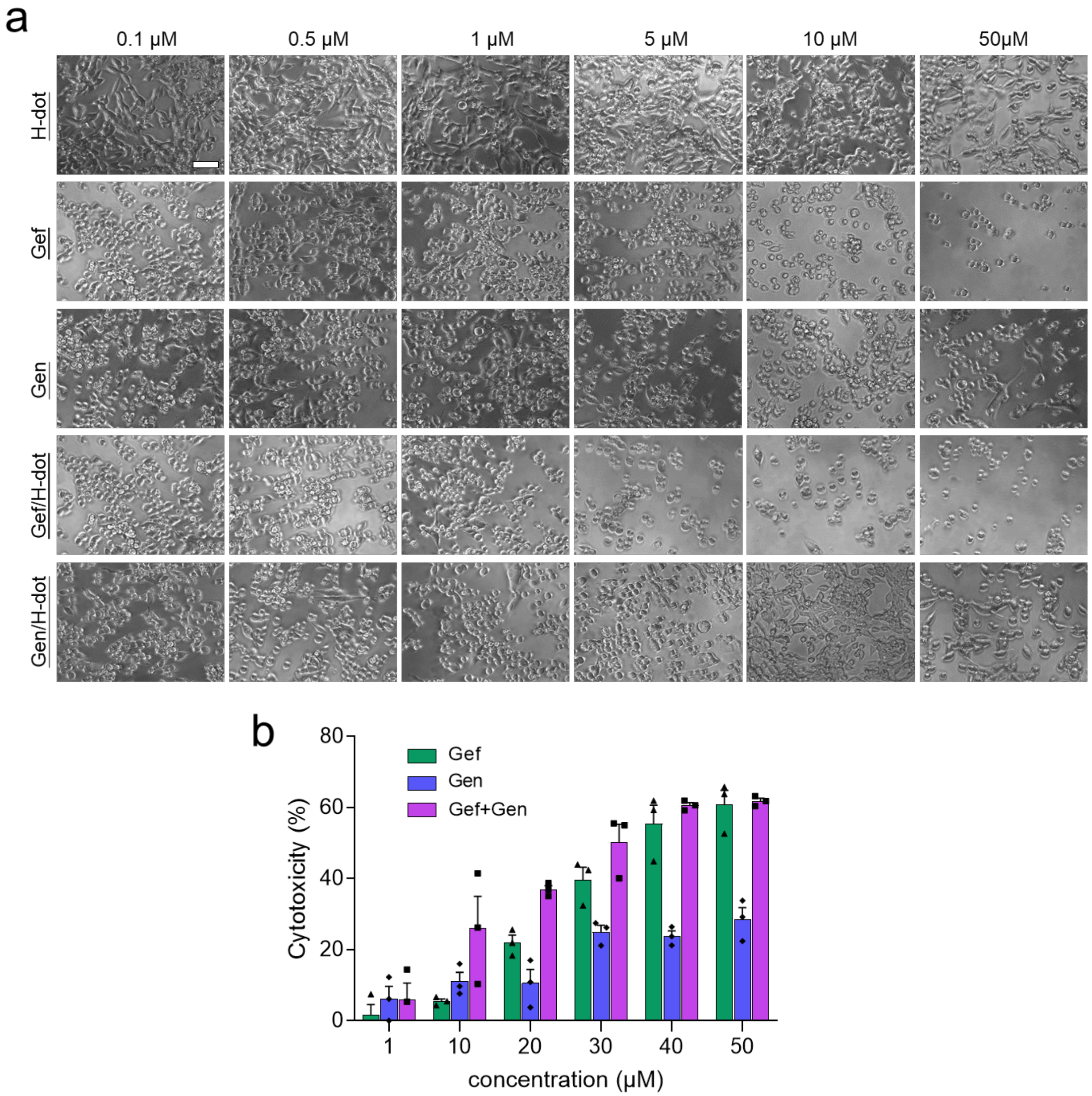

Figure S8. ***In vitro*** **therapeutic efficacy test.** (a) The morphology of LLC cells before and after the treatment with H-dot, Gef, Gen, Gef/H-dot, and Gen/H-dot for 24 h was assessed using microscopic images (20×). Scale bar: 200 μm. (b) Cytotoxicity of Gef, Gen, and Gef+Gen at various concentrations. The combination index (CI) is calculated by using the software of CompuSyn.

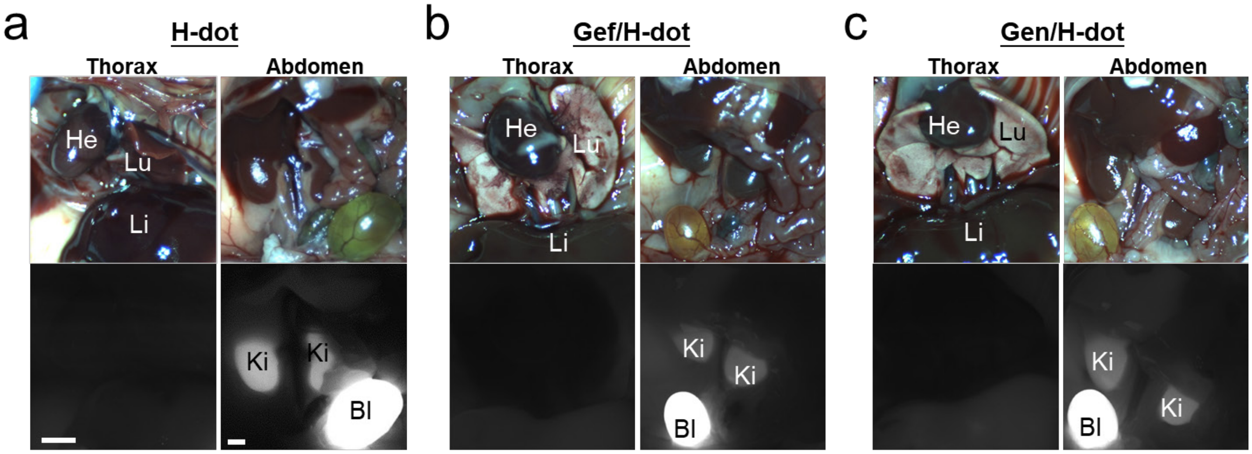

Figure S9. Comparisons of biodistribution of H-dot and drug/H-dot. (A) H-dot, (B) Gef/H-dot, and (C) Gen/H-dot were injected separately into mice, and NIR imaging was carried out at 4 h post-injection. Thorax and abdominal region with a surgical opening. Abbreviations used: Tu, Tumor, Bl, bladder; Du, duodenum; He, heart; In, intestine; Ki, kidneys; Li, liver; Lu, lungs; Mu, muscle; Pa, pancreas; Sp, spleen. Scale bars: 5 mm.

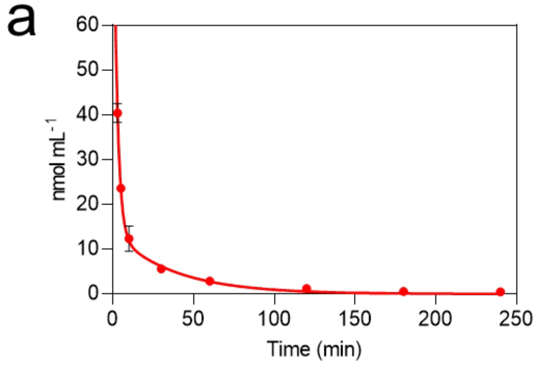

b

Name	H-dot
Molecular weight ($g \cdot mol^{-1}$)	~15,400
Injected dose($nmol \cdot kg^{-1}$)	2500
$t_{½\alpha}$ (min)	1.11 ± 0.03
$t_{½\beta}$ (min)	32.44 ± 13.56
Area under the curve (AUC, $min \cdot nmol \cdot mL^{-1}$)	707.20 ± 36.80
Plasma clearance (CL, $mL \cdot min^{-1}$)	0.08 ± 0.00
Volume of distribution (Vd, $mL \cdot kg^{-1}$)	166.93 ± 71.93

Figure S10. Pharmacokinetics of H-dot. (A) Serum concentration (nmol/mL) decay curve of H-dot. Blood samples from the H-dot injected mice were collected from the tail vein at time points: 1, 3, 5, 10, 30, 60, 120, 180, and 240 min. (B) Pharmacokinetic parameters of H-dot. Mean ± s.e.m. (n = 3 per group).

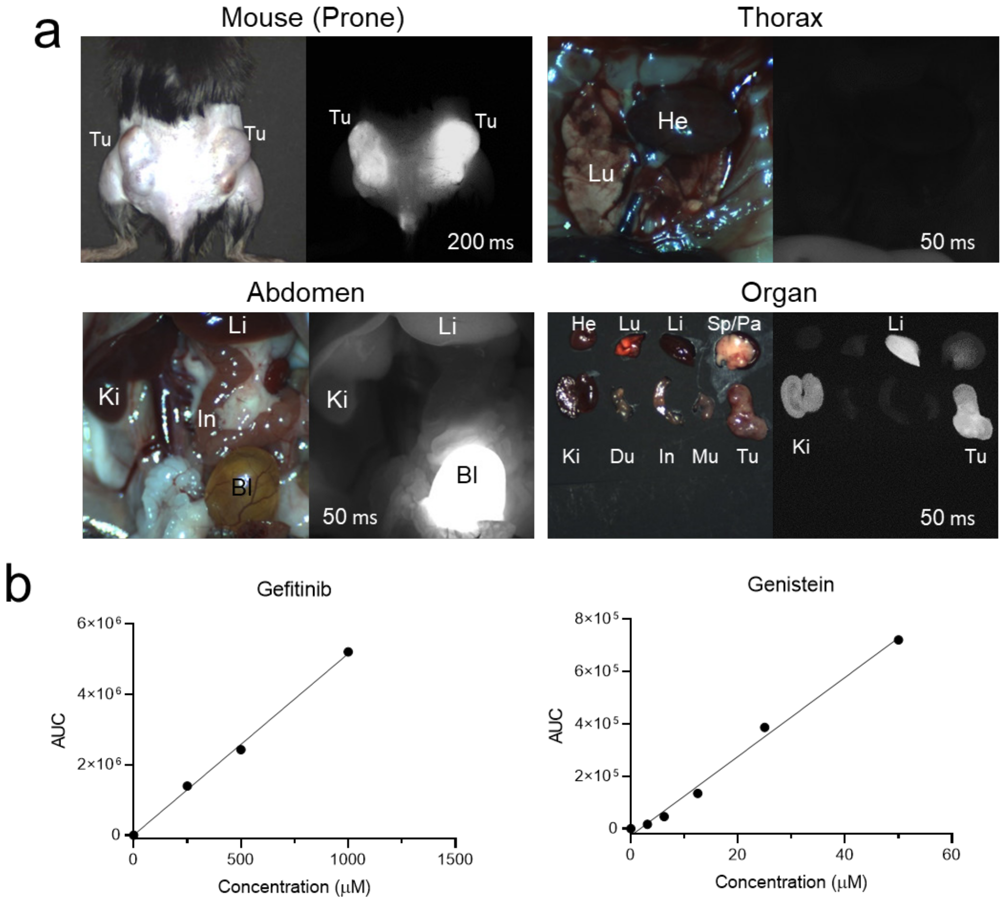

c

	Gef	Gen
%ID	1.7	0.2
%ID/g	6.2	0.7

Figure S11. **Tumor targeting and delivery efficiency of Gef/H-dot+Gen/H-dot.** A) NIR fluorescence images of subcutaneous tumors. Gef/H-dot+Gen/H-dot were injected into lung cancer bearing mice. B) Standard calibration curves of Gef and Gen using HPLC. C) Measured % injection dose (%ID) of Gef and Gen from resected tumor tissues at 24 h post-injection.

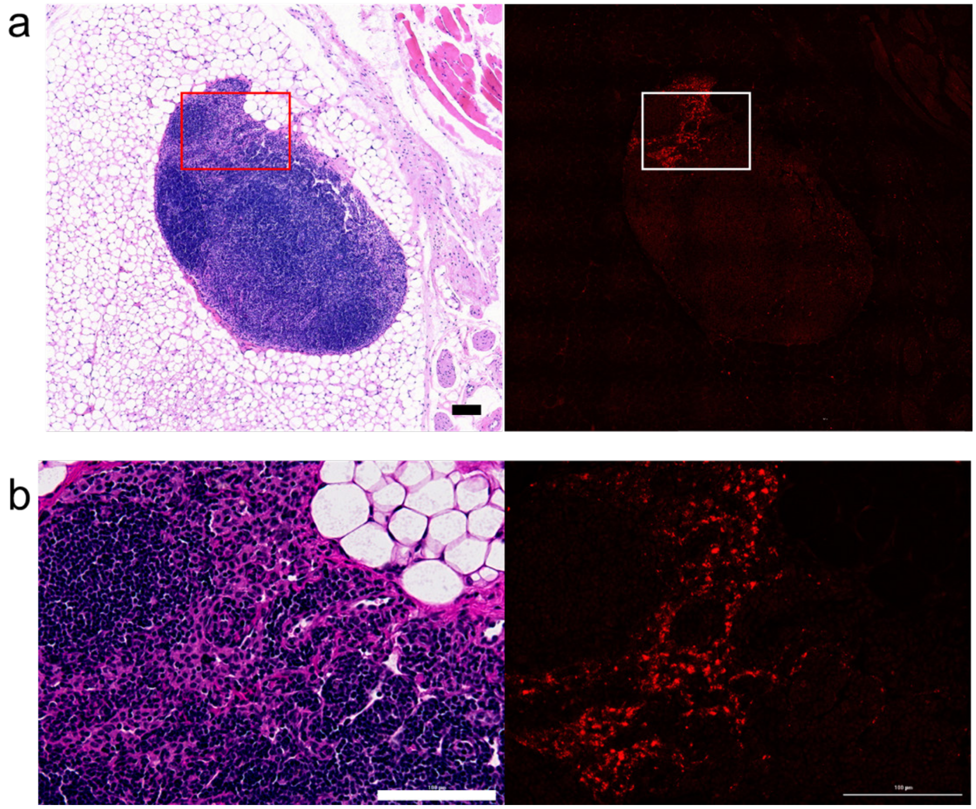

Figure S12. Image-guidance tumor pathology test. NIR fluorescence imaging of LLC lung tumor mice model using Gef/H-dot with Gen/H-dot injected 24 h prior to imaging. Resection of tumors under the guidance of real-time fluorescence imaging, postoperative pathological H&E staining, and pathological detection under the guidance of real-time fluorescence imaging. Scale bars: 100 μm. (A) H&E staining and fluorescence imaging of metastatic lymph nodes (2×). (B) H&E staining and fluorescence imaging of metastatic lymph nodes (20×)

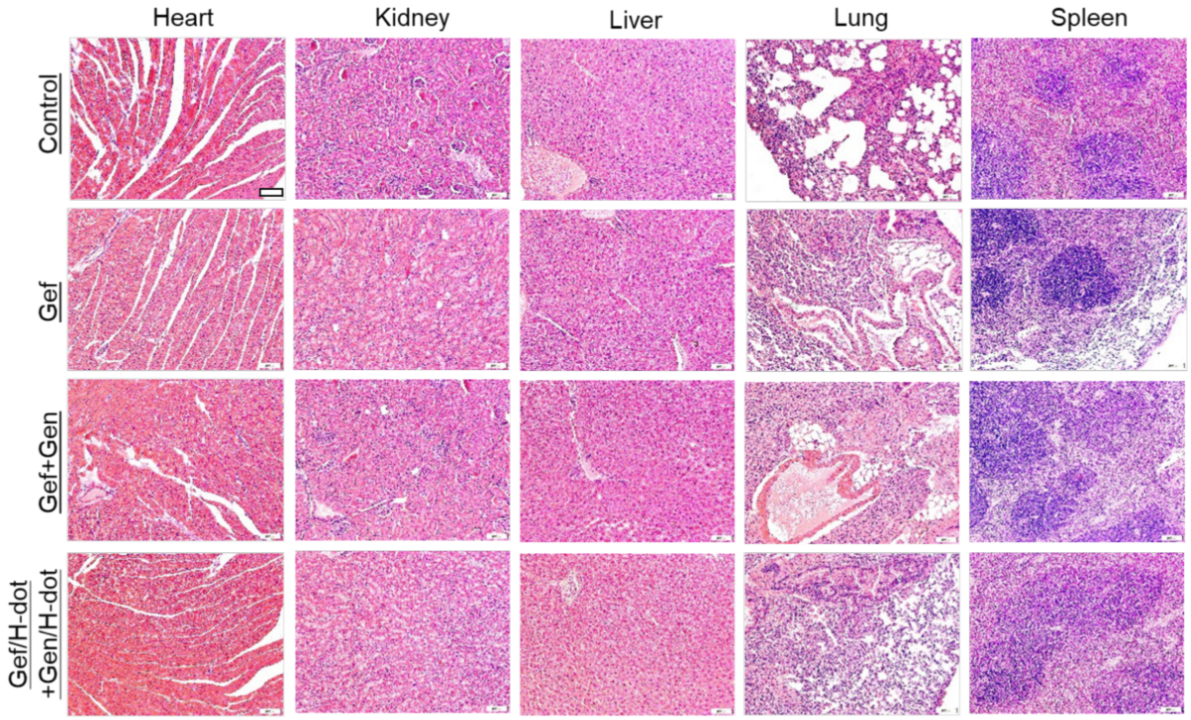

Figure S13. Histological assessment of toxicity on major organs treated with drugs. Twenty-eight B6 mice with LLC subcutaneous tumors were divided into four groups which received saline, Gef, Gef+Gen, and Gef/H-dot+Gen/H-dot respectively 11 times within 14 days (n = 7 for each group). H&E-staining microscope images (10×) of heart, liver, spleen, lung, and kidney from different groups after treatment with different formulations. Scale bar: 100 μm.

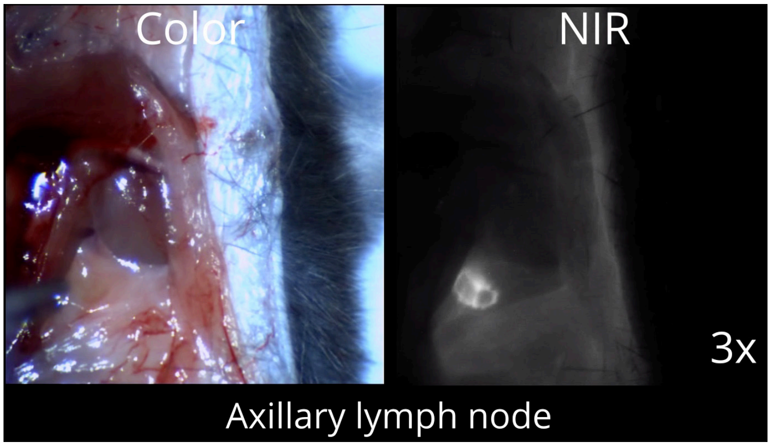

Supplementary Movie S1. Real-time movie of LLC lung tumor-bearing mice injected with Gef/H-dot at 24 h post-injection. Resection of lymph node metastases under the real-time guidance of NIR fluorescence imaging corresponding to **Figure 4C**.
